# Multiple independent origins of auto-pollination in tropical orchids (*Bulbophyllum*) in light of the hypothesis of selfing as an evolutionary dead end

**DOI:** 10.1186/s12862-015-0471-5

**Published:** 2015-09-16

**Authors:** Alexander Gamisch, Gunter Alexander Fischer, Hans Peter Comes

**Affiliations:** Department of Ecology and Evolution, University of Salzburg, A-5020 Salzburg, Austria; Kadoorie Farm and Botanic Garden Corporation, Lam Kam Road, Tai Po, NT Hong Kong

## Abstract

**Background:**

The transition from outcrossing to selfing has long been portrayed as an ‘evolutionary dead end’ because, first, reversals are unlikely and, second, selfing lineages suffer from higher rates of extinction owing to a reduced potential for adaptation and the accumulation of deleterious mutations. We tested these two predictions in a clade of Madagascan *Bulbophyllum* orchids (30 spp.), including eight species where auto-pollinating morphs (i.e., selfers, without a ‘rostellum’) co-exist with their pollinator-dependent conspecifics (i.e., outcrossers, possessing a rostellum). Specifically, we addressed this issue on the basis of a time-calibrated phylogeny by means of ancestral character reconstructions and within the state-dependent evolution framework of BiSSE (Binary State Speciation and Extinction), which allowed jointly estimating rates of transition, speciation, and extinction between outcrossing and selfing.

**Results:**

The eight species capable of selfing occurred in scattered positions across the phylogeny, with two likely originating in the Pliocene (ca. 4.4–3.1 Ma), one in the Early Pleistocene (ca. 2.4 Ma), and five since the mid-Pleistocene (ca. ≤ 1.3 Ma). We infer that this scattered phylogenetic distribution of selfing is best described by models including up to eight independent outcrossing-to-selfing transitions and very low rates of speciation (and either moderate or zero rates of extinction) associated with selfing.

**Conclusions:**

The frequent and irreversible outcrossing-to-selfing transitions in Madagascan *Bulbophyllum* are clearly congruent with the first prediction of the dead end hypothesis. The inability of our study to conclusively reject or support the likewise predicted higher extinction rate in selfing lineages might be explained by a combination of methodological limitations (low statistical power of our BiSSE approach to reliably estimate extinction in small-sized trees) and evolutionary processes (insufficient time elapsed for selfers to go extinct). We suggest that, in these tropical orchids, a simple genetic basis of selfing (via loss of the ‘rostellum’) is needed to explain the strikingly recurrent transitions to selfing, perhaps reflecting rapid response to parallel and novel selective environments over Late Quaternary (≤ 1.3 Ma) time scales.

**Electronic supplementary material:**

The online version of this article (doi:10.1186/s12862-015-0471-5) contains supplementary material, which is available to authorized users.

## Background

Flowering plants (angiosperms) display a bewildering diversity and, in many cases, complexity of mating systems compared to most animal groups [1, 2]. The overwhelming majority of angiosperms are characterized by cross-pollination of their flowers, whereby mechanisms preventing self-fertilization range from the separation of sex functions within flowers (herkogamy, dichogamy) and plants (monoecy), to separate sexes (dioecy), and self-incompatibility (SI) systems [2, 3]. Still, about 20–25 % of all flowering plant species are predominantly selfing [4], which is commonly thought to reflect two main advantages, which are not necessarily mutually exclusive. First, selfing genes should enjoy a two-fold transmission advantage over genes for outcrossing [5–7] and, second, the loss or paucity of pollinators or mates should selectively favour selfing individuals/populations because it provides reproductive assurance [8–10]. On the other hand, inbreeding depression has long been recognized to counteract the evolution of selfing and explaining the maintenance of outcrossing [11–13]. In fact, selfing lineages or taxa are typically assumed to be an evolutionary ‘blind alley’, or ‘dead end’ ([14–17]; selfing as an evolutionary dead end or SEDE hypothesis), presumably because of their limited capacity to adapt to changing environment, or their susceptibility to the accumulation of deleterious mutations [2, 7, 14]. Taking a macroevolutionary perspective, however, our body of knowledge is still limited regarding the historical evolutionary dynamics of mating system transitions among closely related taxa differing in outcrossing vs. selfing strategies and over extended time scales.

Several underlying assumptions of the SEDE hypothesis, as originally proposed by Stebbins [15, 16], have turned out to be more complex than initially thought. Nonetheless, there is wide consensus about two major predictions: (1) evolutionary change is unidirectional and irreversible from outcrossing to selfing; and (2) selfing lineages suffer from an increased risk of extinction owing to a reduced potential for adaptation [7, 14, 17]. Consistent with both connotations, several molecular phylogenies have inferred outcrossing systems to be ancestral, with selfing species commonly located at terminal branches [18–23]. However, the historical dynamics of outcrossing-to-selfing transitions are often difficult to reconstruct and a few studies have even pointed out the possibility of reversals [21, 24–29]. Moreover, potential departures from theory are generally suspected to result from methodological difficulties associated with, for example, phylogenetic estimation *per se*, incomplete or missing taxonomic accounts, ambiguous character state coding, and/or subjectively chosen character transformation weights [17, 24, 25, 30, 31]. Other challenges are posed by the presence of intraspecific mating type polymorphisms (e.g., [25]), and possible confounding effects of diversification and character state evolution [28, 32, 33]. Perhaps most strikingly, the second claim of the SEDE hypothesis, concerning the assumed higher extinction [viz. negative net diversification (speciation minus extinction) rate] associated with selfing, has largely been ignored in the literature [14], possibly because of the difficulties of obtaining accurate and unbiased estimates of extinction rates from molecular phylogenies [33–35].

However, recent developments of model-based methods of phylogenetic inference, molecular dating, and ancestral character state reconstructions [36, 37] should overcome at least some of the past methodological obstacles encountered in phylogenetic studies of mating type evolution. More reliable tests of the SEDE hypothesis are also expected from a recently developed, yet sparingly employed tree-based model of character evolution (Binary State Speciation and Extinction [BiSSE]; [33, 38]) that allows the joint estimation of rates of binary character change as well as state-dependent rates of extinction and speciation [39–41]. By capitalizing on these recent advances, the present study investigates mating type shifts (outcrossed vs. self-fertilized) during the diversification of a well-defined clade of epidendroid orchids of the genus *Bulbophyllum*Thouars from Madagascar and adjacent islands. No study to date has subjected members of Orchidaceae to such kind of macroevolutionary analysis, and there are several reasons detailed below why this group of tropical orchids provides a particular interesting system for the study of selfing evolution.

**Fig. 1:**
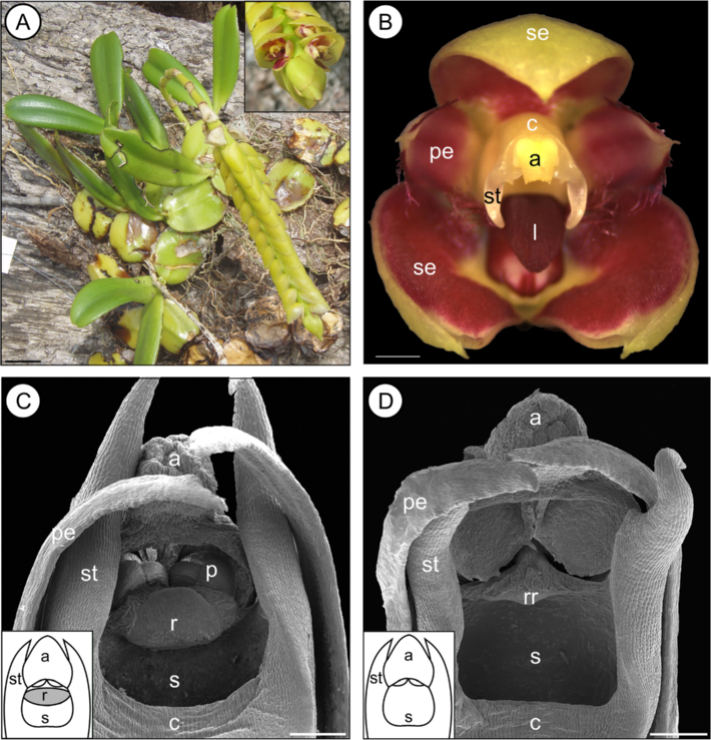
Eight species of Madagascan *Bulbophyllum* clade C vary intra-specifically for mating type. **a** Habit and detail of inflorescene (inset) and (**b**) flower close up of a member of Madagascan *Bulbophyllum* clade C (*B. cirrhoglossum*). **c** Outcrossing individuals (Type I) differ from their (**d**) auto-pollinating conspecifics (Type II) by the presence of the rostellum. Note that the pollinia in (**d**) were lost during sample preparation. Abbreviations: a, anther; c, column (gynostemium); l, labellum; p, pollinia; pe, petal; r, rostellum; rr rudimentary rostellum; s, stigma; se, sepal; st, stelidium. Scale bars: A = 20 mm; B = 1 mm; C, D = 0.1 mm. Photographs by A. Sieder (**a**) and A. Gamisch (**b**). The habitus photograph (**a**) and the scanning electron microscopy micrographs of *B. quadrifarium* and sketches of the different gynostemium morphs (**c**, **d**) are modified from [48]

Most species of *Bulbophyllum* (ca. 2400 spp.; [42]) are tropical epiphytes, self-compatible, and adapted to cross-pollination by flies or more rarely bees [43, 44], with flowers typically possessing an erect, non-receptive ‘rostellum’. This is a shelf-like projection of the column (i.e., the united androecium/gynoecium of orchids), which separates the single anther from the stigmatic cavity below, thereby preventing self-fertilization without the aid of a vector (i.e., auto-pollination; *sensu* [45]) and aiding in gluing the pollinia to the pollinator [43, 46]. However, recent micro-morphological and experimental studies in *Bulbophyllum* from Madagascar and adjacent islands [47, 48] have identified eight species that vary intra-specifically for rostellum and thus mating type. In detail, auto-pollinating individuals (or ‘morphs’) differ from their outcrossing conspecifics (Type I) in the lack of the rostellum (Type II; see Fig. 1) or, in case of a single species, in displaying a sub-erect rostellum with stigmatic function (Type III; [47, 48]). These dimorphic species belong to a taxonomically and phylogenetically well-circumscribed clade (henceforth, ‘clade C’) comprising sects. *Calamaria*, *Humblotiorchis*, and *Bifalcula* [49, 50]; Fischer et al., unpubl. data). All remaining species of this lineage, as well as, representatives of related sections studied so far, are monomorphic for the outcrossing rostellum type [48]. Although the precise genetic control and heritability of Types I–III remain obscure, each morph is presumed to be genetically encoded and unresponsive to environmental influences, as within-individual variation in mating type was not observed under controlled conditions [47, 48].

Observing a relatively high incidence of auto-pollination in *Bulbophyllum* is rather unexpected because this genus has long been considered to be highly pollinator-dependent (see above). Even though estimates of auto-pollination in Orchidaceae as a whole (ca. 25,000 spp.) are relatively high with about 31 % of species [51, 52], auto-pollination is generally considered to be rare in species from the tropics [45, 53], excepting small islands (e.g., La Réunion; [54]). When compared to other earlier phylogenetic studies of selfing evolution [7, 20, 21, 26–28, 55, 56], our study system is notable in at least two respects: (1) selfing is not achieved through the breakdown of a floral multi-organ (e.g., heterostyly) or genetic (e.g., SI) mechanism associated with outcrossing but rather due to a structural, discrete-state character transition affecting floral micro-morphology (i.e., rostellum presence/absence); and (2) selfing morphs (‘selfers’) apparently only coexist with their pollinator-dependent conspecifics (‘outcrossers’), i.e., selfing is never fixed within species. Because conspecific individuals differing in morph-type are virtually identical in overall (floral, vegetative) phenotype [47, 48], selfing may have evolved only recently and multiple times within clade C. This hypothesis, however, has yet to be tested since phylogenetic relationships within clade C still remain obscure due to incomplete taxon and gene sampling in an earlier phylogenetic analysis [50].

The overall aim of this study was to examine the polarity, frequencies, time scales, and consequences of mating type transitions during the diversification of clade C in order to test major predictions raised by the SEDE hypothesis, i.e., irreversibility of transitions to selfing and high extinction rate of selfing species. Our first aim was to infer a robust, fossil-calibrated phylogeny of clade C using DNA sequence data (five plastid and three nuclear loci) to explore the timing and tempo of species diversification. Second, we reconstructed ancestral character states onto the phylogenetic tree to evaluate when and how often transitions occurred between outcrossing and ‘selfing’ (viz. dimorphic) mating types, and whether uni- or bi-directionally. Finally, we used the state-dependent BiSSE model of character evolution to recover the processes proposed by the SEDE hypothesis, and to test in particular whether the evolution of selfing in these tropical orchids was associated with higher levels of extinction. Overall, this study is the first to demonstrate within a macroevolutionary framework that, contrary to prevailing views, selfing not always needs to be an endpoint of mating type evolution in flowering plants, suggesting instead more frequent origins of this character than previously thought.

## Methods

### Study system and mating system categorization

The most recent taxonomic treatment of sects. *Calamaria*, *Humblotiorchis*, and *Bifalcula* recognizes 32 species ([57]; Fischer et al., unpubl. data). Fifteen of those species were included in the study of Madagascan *Bulbophyllum* conducted by [50], using a combined sequence matrix of one nuclear ribosomal (ITS) and four plastid gene regions, and were identified as a monophyletic group (‘clade C’; Bayesian posterior probability [PP] of 0.93), albeit with poorly resolved interspecies relationships.

**Fig. 2:**
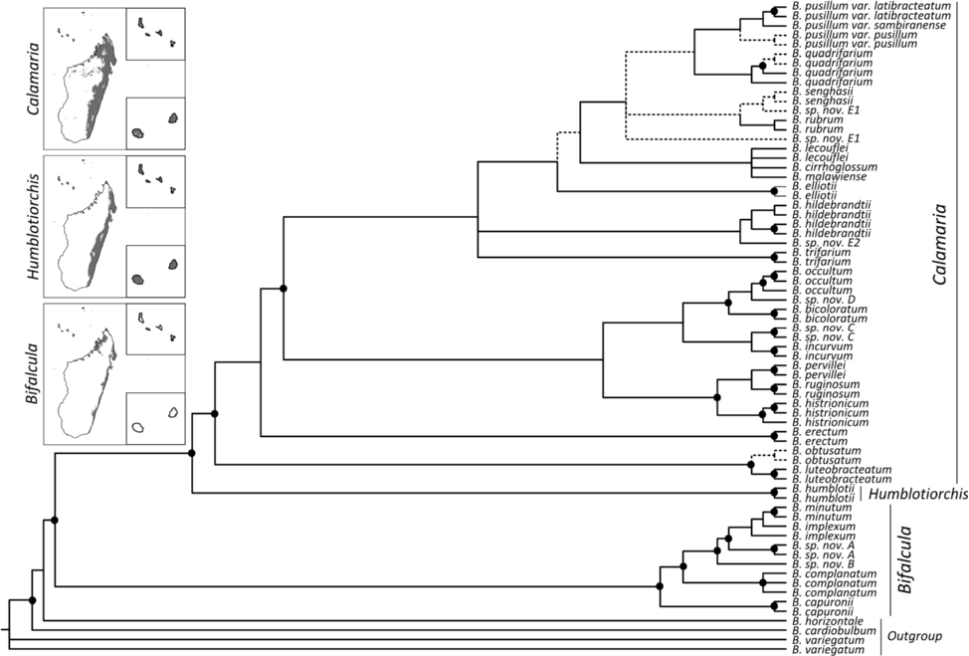
Majority-rule consensus of Madagascan *Bulbophyllum* clade C (sects. *Calamaria*, *Humblotiorchis*, *Bifalcula*) estimated under Bayesian Inference (BI) for the eight-gene dataset (nrITS, *PEPC*, *PI*, and five plastid regions combined). Solid dots indicate nodes with Bayesian posterior probability (PP) of 1 and parsimony bootstrap percentage (BP) ≥ 85. Branches only weakly supported by Bayesian analysis (PP ≤ 0.95) are indicated through stippled lines. The maps on the left show the potential current distribution of each section in Madagascar and adjacent islands (upper inset: Comores; lower inset: La Réunion, Mauritius) based on bioclimatic variables (Gamisch et al. unpubl. data; see [Additional file 1] for details on the distribution of each species)

Most species of clade C are leaf-succulent epiphytes restricted to Madagascar (27 spp.) and/or adjacent islands (Mascarenes: La Réunion/Mauritius, Comores, Seychelles; 3 spp.), with the remainder found in Madagascar and/or the East African mainland (*B. humblotii*, *B. malawiense*; see maps (insets) of Fig. 2 and [Additional file 1)). Their preferential habitats include seasonally dry to humid evergreen forests, or more rarely marshland, at various altitudes (0–1800 m) ([42, 57, 58] Fischer et al., unpubl. data). A list of clade C species, together with their sectional affiliation and geographical distribution, is provided in the [Additional file 1].

Recent micro-morphological and experimental studies [47, 48] revealed that mating type in clade C is directly related to column (gynostemium) type, most often involving the presence or absence of the rostellum. In detail, of the 29 species analysed by [48] (1–25 individuals per species; mean ± SD: 7.17 ± 6.11; 208 individuals in total, 21 species were found to possess only pollinator-dependent individuals (‘outcrossers’) with the conventional (erect) rostellum (Type I); by contrast, seven species proved to be dimorphic for mating type, comprising both Type I individuals and others (Type II) capable of vector-less, autonomous fruit set (‘selfers’) due to the lack of the rostellum (Fig. 1). Moreover, a single species (*B. bicoloratum*) was found to have three mating types by additionally possessing selfing individuals with a receptive (sub-erect) rostellum (Type III; see also [47]).

We use the per-species mating type classification of [48] as a basis for reconstructing the history of selfing evolution in clade C presented here (Table 1). Notably, for two species (i.e., *B. bicoloratum*, *B. occultum*), there is morphological (rostellum-type) as well as population genetic (DNA fingerprint, microsatellite) and reproductive (natural fruit set) evidence available (based on ca. 200 individuals in total) suggesting that selfing individuals are indeed frequent enough to be thought of representative for the whole species ([47, 48]; U. Jaros, unpubl. data). As regards the other six species likewise designated as ‘selfing’ (i.e., *B. complanatum*, *B. erectum*, *B. pusillum*, *B. obtusatum*, *B. quadrifarium*, *B. humblotii*), selfing individuals are probably also common, if not predominant, although this inference is based on morphological evidence only and a more limited sampling (i.e., among 62 individuals surveyed in total, 32 were found lacking a rostellum; [48]). Hence, all currently available data point to selfing as the predominant mode of reproduction in the eight species designated here as ‘selfing’ (Table 1).

**Table 1:** Coding of mating types for 30 *Bulbophyllum* clade C species included in this study, following the mating type classification of [48]. Species fixed for outcrossing were coded as state ‘0’, and dimorphic (outcrossing/selfing) species as state ‘1’ (‘any-instance’ coding; see text for details). For two species (*B*. spp. nov. ‘*B*’ and ‘*D*’) mating type was coded as missing (‘NA’)

Section/species	Mating type	Character coding	
*Bifalcula*			
*B. capuronii* Bosser	Outcrossing		0
*B. complanatum* H.Perrier	Dimorphic		1
*B. implexum* Jum. & H.Perrier.	Outcrossing		0
*B. minutum* Thouars	Outcrossing		0
*B.* sp. nov. *‘A’*	Outcrossing		0
*B.* sp. nov*. ‘B’*	Unknown		NA
*Calamaria*			
*B. bicoloratum S*chltr.	Trimorphic		1
*B. cirrhoglossum* H.Perrier	Outcrossing		0
*B. elliottii* Rolfe	Outcrossing		0
*B. erectum* Thouars	Dimorphic		1
*B. hildebrandtii* Rchb.f.	Outcrossing		0
*B. histrionicum* G.A.Fischer & P.J.Cribb	Outcrossing		0
*B. incurvum* Thouars	Outcrossing		0
*B. lecouflei* Bosser	Outcrossing		0
*B. luteobracteatum* Jum. & H.Perrier	Outcrossing		0
*B. malawiense* B.Morris	Outcrossing		0
*B. obtusatum* (Jum. & H.Perrier) Schltr.	Dimorphic		1
*B. occultum* Thouars	Dimorphic		1
*B. pervillei* Rolfe	Outcrossing		0
*B. pusillum* (H.Perrier) G.A.Fischer & P.J.Cribb	Dimorphic		1
*B. quadrifarium* Rolfe	Dimorphic		1
*B. rubrum* Jum. & H.Perrier	Outcrossing		0
*B. ruginosum* H.Perrier	Outcrossing		0
*B. senghasii* G.A.Fischer & A.Sieder	Outcrossing		0
*B. trifarium* Rolfe	Outcrossing		0
*B.* sp. nov. *‘C’*	Outcrossing		0
*B.* sp. nov. *‘D’*	Unknown		NA
*B.* sp. nov. *‘E1’*	Outcrossing		0
*B.* sp. nov. *‘E2’*	Outcrossing		0
*Humblotiorchis*			
*B. humblotii* Rolfe	Dimorphic		1

The BiSSE method used in the present study (detailed below) requires that the states that are compared must be coded as a binary character [21, 39, 40]. If species exhibit multiple (‘polymorphic’) character states, the most common state is usually taken as the only one present (e.g., [21, 40, 56]). However, such a frequency-dependent coding strategy is prone to sampling bias if sample sizes of character statement assessment are low [59], as in the present study. To accommodate this latter problem, we used the ‘any instance’ method (i.e., fixation of the ancestral state = 0; and polymorphism or fixation of the derived state = 1; [59, 60]) and coded the mating types of clade C species ([48]; Table 1) as being either outcrossing (0) or selfing (1), and thus regardless of the fact that selfing species also harbour outcrossing morphs. Following [59–61], we justify this ‘any-instance’ coding, as (1) population-level estimates of morph frequencies are mostly lacking for these tropical orchids (see above); and (2) some assessment of selfing as a derived condition in clade C can be made *a priori* by comparison with other Madagascan *Bulbophyllum* species used as outgroups [47, 48].

### Taxon sampling and DNA sequencing

Thirty species (65 accessions) out of the 32 species ascribed to sects. *Calamaria* (23/25 spp.), *Humblotiorchis* (1/1 sp.), and *Bifalcula* (6/6 spp.) were included in our study [Additional file 1). This taxon sampling comprises 15 species that were not sampled in [50], six yet undescribed species (‘spp. nov.’), and 28 species with known mating type, including eight selfers (Table 1). We were unable to obtain material for DNA extraction from the two remaining species of sect. *Calamaria*, i.e., *B. sp*. ‘*F*’ (with unknown mating type) and *B. cryptostachium* (Type I; [48]). We also included, as outgroups, one species each from sects. *Kainochilus*, *Inversiflorum*, and *Alcistachys*, whose close relationship to clade C is supported on molecular and morphological grounds [49, 50]. Like all extra-clade C species of Madagascan *Bulbophyllum*, these outgroups have so far proved to be monomorphic for the outcrossing mating type [48].

Most *Bulbophyllum* samples used in this study were field-collected in Madagascar, with all necessary permits obtained by the Département des Eaux et Fôrets (Madagascar), or received from botanic gardens (Parc Botanique et Zoologique de Tsimbazaza; Parc National de La Réunion; Vienna; Salzburg) and herbaria (REU, SZU, TAN, WU). Additional DNA samples were obtained from the Jodrell Laboratories, Kew. Whole genomic DNA was extracted from fresh, silica-dried leaf material using the 2x CTAB method [62]. A few accessions, preserved as leaf extract on FTA® Mini Cards, were processed using the FTA® Starter Pack (Whatman, Florham Park, USA) according to the manufacturer’s specifications.

We PCR amplified and sequenced five plastid and three nuclear gene regions. The former comprised four intergenic spacers (*atp*I*–atp*H, *psb*A*–trn*H, *trn*D–*trn*E, *trn*T*–trn*S [including the *rps*4 gene]) and a hypothetical open reading frame gene region (*ycf1*). Nuclear regions were the internal transcribed spacer (ITS) region of ribosomal DNA (including the 5.8S gene) and portions of two low-copy genes, i.e., the structural gene encoding phosphoenolpyruvate carboxylase (*PEPC*; [63]), and the floral homoeotic gene *pistillata/globosa* (*PI*; [64]). For amplification we used protocols and primers from [50] for ITS, *psb*A*–trn*H, and *trn*D–*trn*E, [65, 66]) for *atp*I*–atp*H and *trn*T*–trn*S, and [67] for *ycf1*. Details of the newly designed primers for *PEPC* and *PI* are provided in [Additional file 2]. Amplification products were sequenced at Macrogen Inc. (Seoul, South Korea) using the above PCR primers. Sequences were edited and aligned using the MUSCLE algorithm with default settings as implemented in Geneious version 5.4 [68]. For all plastid and nuclear regions, sequence length variation among species was low. Hence, gaps were generally treated as missing data. GenBank sequence accession numbers and voucher details are summarized in [Additional file 1). We included outgroups and multiple intra-specific samples in the phylogenetic analyses and removed outgroups for the dating analysis in Beast version 1.6.1 [36] (described further below), but removed each of those in all subsequent Beast, diversification, and mating-type transition analyses, resulting in a total of 30 terminal species. For these latter analyses, multiple accessions of the same species were pruned by keeping the most early-branching accession.

### Phylogenetic inference

Four data partitions were defined, corresponding to each nuclear dataset (ITS, *PEPC*, *PI*) and the five plastid regions combined. We identified the best-fit models of nucleotide substitution for each partition using the Akaike Information Criterion (AIC) in jModeltest version 0.1.1 [69], i.e., the GTR + G model for the ITS and plastid datasets and the HKY + G model for *PEPC* and *PI*. These models were then applied to the phylogenetic analyses of each partition using Bayesian Inference (BI) as implemented in MrBayes version 3.1.2 [70], with *B. variegatum* (sect. *Alcistachys*) assigned as outgroup (note, MrBayes allows for only one outgroup sample). The Markov chain Monte Carlo (MCMC) algorithm was run with two independent runs of four Metropolis-coupled chains with 20 million generations each, with trees sampled every 1,000 generations. After assessing convergence between runs by monitoring the standard deviation of split frequencies in MrBayes and using the effective sampling size (ESS > 200) criterion in Tracer version 1.5 [71], the first 25 % of sampled trees were discarded as burn-in (based on stabilization of the log-likelihood of the trees). A 50 % majority-rule consensus tree was constructed from the remaining 15,000 trees to estimate posterior probability values for each node. For comparison, node support was also estimated by bootstrapping under maximum parsimony (MP) in Paup* version 4.0b10 [72] using 1000 heuristic search replicates and the following settings: 100 random addition sequence replicates with three trees held at each step, tree bisection reconnection (TBR) branch swapping, and saving no more than 10 trees per replicate. The degree of incongruence among the four data partitions was checked by looking for conflicting clades that were strongly supported in terms of both Bayesian posterior probability (PP ≥ 0.95) and parsimony bootstrap percentage (BP ≥ 85) (see also [73, 74]). Since no such incongruence was detected see [Additional files 3, 4, 5 and 6], we concatenated all partitions into a single alignment, and re-analysed it under the above BI and MP settings.

### Bayesian hypothesis testing of monophyletic selfers

To test whether selfing species comprise one or more monophyletic lineages, we used logarithmic Bayes Factor (BF) comparisons [71, 75, 76] to evaluate the support for different topological scenarios in clade C based on the combined (nuclear/plastid) BI tree (Fig. 2). Essentially, this analysis helped to provide an initial assessment of topologically independent shifts in mating type. We used Tracer to compare the marginal tree likelihoods of models constrained for monophyly of selfers (i.e., by constraining all selfers of, respectively, clade C, sects. *Calamaria* + *Humblotiorchis*, and sect. *Calamaria*), with those obtained under the unconstrained (alternative) hypothesis (see Table 2). For model comparison, we used the BF ‘test statistic’ of 2 log(marginal likelihood [unconstrained model]) – log(marginal likelihood [constrained model]). Following [75], evidence against the constrained model (i.e., the null hypothesis, H_0_) was considered to be ‘positive’ (BF value = 2–6), ‘strong’ (6–10), or ‘very strong’ (> 10).

**Table 2:** Results of the monophyly tests for selfing *Bulbophyllum* clade C species using logarithmic Bayes Factor (BF) comparisons

Monophyly constraint	Marginal tree likelihood	+/- SE	2(log BF)
No constraint	-15887.6	+/- 0.301	-
All selfers of clade C	-16563.5	+/- 0.317	676.0
All selfers of sects. *Calamaria* + *Humblotiorchis*	-16400.8	+/- 0.308	513.2
All selfers of sect. *Calamaria*	-16371.7	+/- 0.348	484.1

### Evolutionary transitions in mating types

Based on the data of mating type (Table 1), we reconstructed transitions between outcrossing and selfing mating types in clade C using a two rate (Mk2) model of character evolution in the Bayesian (MCMC) framework of the submodule Multistate of BayesTraits version 1.0 ([77]; http://www.evolution.rdg.ac.uk). Following [59–61], we designated the species as outcrossing or selfing using the ‘any-instance’ coding method (see above). Mating types were traced over the last 5,000 post-burn-in trees of the BI analysis to incorporate branch length and topology uncertainty. The MCMC analyses were run during 5,050,000 generations, with a *ratedev* parameter of 110 (obtained from initial test runs), a reversible-jump hyperprior with an exponential prior (mean seeded from a uniform distribution on the interval 0 to 30), and a burn-in of 50,000 generations.

**Fig. 3:**
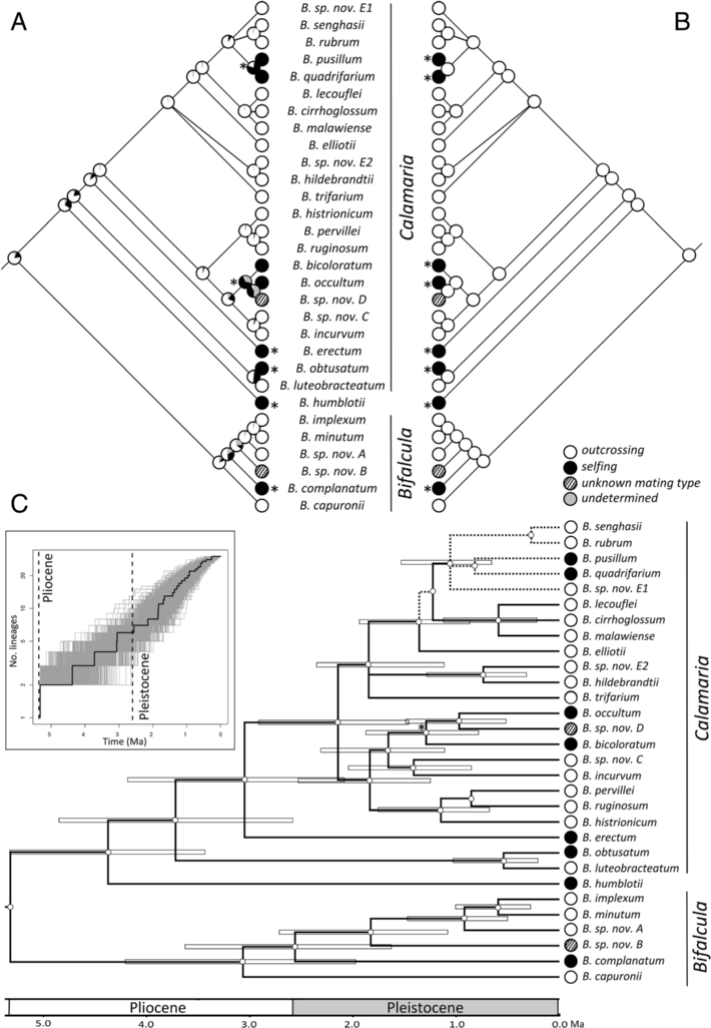
Ancestral state reconstructions of mating type and chronogram of Madagascan *Bulbophyllum* clade C. **a** Reconstruction under an unconstrained Mk2 model using Bayestraits. **b** Maximum likelihood (ML) reconstruction under the best-fitting BiSSE model (see also Table 4). Pies at internal nodes indicate mean relative posterior probabilities assigned to alternative states based on 5,000 BI (**a**) or 1,000 Beast trees (**b**). Transitions in mating type are marked by an asterisk. **c** Branch lengths proportional to time (million years ago [Ma]) estimated with a relaxed clock approach (Beast) and summarized onto the topology of the majority-rule consensus of the Bayesian analysis. Divergence dates were inferred using both fossil-based and biogeographic (island age) calibration points (see [Additional files 7, 8, 9, 10, 11, 12 and 13]). Branches only weakly supported by Bayesian analysis (PP ≤ 0.95) are indicated through stippled lines. Horizontal bars indicate 95 % highest posterior density (HPD) for node ages. Also shown are ancestral state reconstructions of mating type under the model-based (BiSSE) reconstructions, with all internal nodes (small open dots) reconstructed as outcrossing. In all probability, selfing evolved independently at five times within clade C since about the mid-Pleistocene (ca. ≤ 1.3 Ma; see node marked by an asterisk). The inset shows a log-lineage-through-time (LTT) plot for Madagascan *Bulbophyllum* clade C, based on the chronogram (black line) and 1,000 trees sampled from the posterior distribution of the Beast analyses (*gray lines*)

Because Mk2 models cannot account for the effect of the character on rates of speciation and extinction [14, 37, 78], we complemented our ancestral character reconstructions by obtaining marginal estimates of ancestral node states over 1,000 Beast trees under our best fitting BiSSE model using maximum likelihood (ML) reconstructions (see [79] for a similar approach). For each method employed, ancestral character states were plotted onto the 50 % majority-rule consensus tree of the BI analysis (Fig. 3a, b). The ML reconstructions of the best fitting BiSSE model were also plotted on the chronogram (Fig. 3c).

### Estimating times of divergence

Estimation of divergence times of clade C was performed in Beast with outgroup taxa removed and using the same unlinked gene partitions and substitution models as in the BI analysis (see above). A birth-death (BD) speciation model was specified as tree prior and an uncorrelated lognormal relaxed clock was assumed [80]. Five independent MCMC runs were performed with 20 million generations each, sampling every 2,000 generations. Following the removal of a conservative burn-in of 15 %, the MCMC samples were combined using LogCombiner version 1.6.1 [36] and inspected in Tracer to confirm convergence of the chain to stationary and assess sampling adequacy (ESS > 200). The results of the Beast analysis were summarized with TreeAnnotator version 1.6.1 [36]. The remaining 42,500 trees were summarized onto the topology of 50% majority-rule consensus tree of the BI analysis with a threshold of zero for the posterior probability of clades and with node heights estimated using median values. The resulting chronogram (Fig. 3c) was visualized in Figtree version 1.3.1 (http://tree.bio.ed.ac.uk/software/figtree/). As there are no fossils of *Bulbophyllum* that can be used to assess the timing of divergence of clade C, we used a multi-step secondary calibration approach based on three fossils of other orchid genera and two geological calibration points taken from island ages. See [Additional file 7] for full details.

### Course and mode of diversification

To visualize how lineages accumulate within clade C through time, we used the R package Ape [81] to derive log-lineage-through-time (LTT) plots from both the chronogram (Fig. 3c) and 1,000 dated Beast trees resampled at a lower frequency in LogCombiner. Under the rate-constant BD model with speciation (*λ*) and extinction rate (*μ*) being constant and *μ* > 0, we would expect a near-straight line whose slope approximately estimates *r* (= *λ* – *μ*) (i.e., the net per lineage diversification rate), but with a slight upturn in the number of lineages near the present with slope ≈ *λ* [82]. Based on the chronogram, we also used birth-death likelihood (BDL) analysis as implemented in Laser version 2.3 [83] to test the fit of clade C to two rate-constant diversification models (pure-birth [PB or Yule]: *μ* = 0; BD: *μ* > 0) against four rate-variable models (see Table 3): the Yule model with two (Y2R) or three (YR3) rates, and two diversity-dependent models with either linear (DDL) or exponential (DDX) diversification [84]. We compared the AIC scores of the best rate-constant model (AIC_RC_) and the best rate-variable model (AIC_RV_) by computing the test statistic ∆AIC_RC_ = AIC_RC_ – AIC_RV_ [83, 85]. Critical values of ∆AIC_RC_ were assessed by simulating 1,000 trees of 30 species under the PB model (null hypothesis) in Laser (function ‘yulesim’), and by comparing the original value against the distribution from simulations [86].

**Table 3:** Results of fitting rate-constant and rate-variable diversification models to the maximum clade credibility chronogram of *Bulbophyllum* clade C (Fig. 3c) using birth-death likelihood (BDL) analysis

Model	*r*_1_	*r*_2_	*r*_3_	*st*_1_	*st*_2_	*a*	*x*	*k*	Ln L	AIC	ΔAIC
PB	0.53	–	–	–	–	–	–	–	25.485	-48.97	3.250
BD	0.53	–	–	–	–	0	–	–	25.485	-46.97	5.250
DDX	0.83	–	–	–	–	–	0.173	–	25.753	-47.505	4.714
DDL	0.87	–	–	–	–	–	–	45.605	26.638	-49.275	2.945
Y2R	0.646	0.159	–	0.43	–	–	–	–	28.221	-50.442	1.778
**YR3**	**0.41**	**8.116**	**0.502**	**1.866**	**1.824**	**–**	**–**	**–**	**31.11**	**-52.22**	**0**

The best fitting model based on the AIC is in bold

*r* = net diversification rate (speciation events per million years), *st* = time (breakpoint) of rate shift, *a* = extinction fraction, *x* = *x*-parameter from the DDX model, *k* = *k*-parameter from the DDL model, Ln L = log-likelihood, AIC = Akaike Information Criterion, ΔAIC = change in AIC relative to the best model

### Estimates of state-dependent diversification/transitions rates

We used the binary-state speciation and extinction (BiSSE) model in the R package DiversiTree version 0.9–2 [38] to estimate (i) state-dependent rates of speciation (i.e., *λ*_O_, *λ*_S_) and extinction (*μ*_O_, *μ*_S_) for clade C species with outcrossing (subscript ‘O’) and selfing (‘S’) mating types, with respective net diversification rates (*r*) derived as *r* = *λ–μ* [33]; and (ii) rates of transition from outcrossing to selfing (*q*_OS_) and vice versa (*q*_SO_). We compared the fit of the full (six-parameter) BiSSE model with unconstrained parameters (i.e., *λ*, *μ*, and *q* allowed to vary) to (i) three models with these parameters constrained to be equal (i.e., *λ*_O_ = *λ*_S_; *μ*_O_ = *μ*_S_; *q*_OS_ = *q*_SO_); (ii) six models with one parameter each fixed to zero (i.e., *λ*_O_, *λ*_S_, *μ*_O_, *μ*_S_, *q*_OS_, or *q*_SO_ = 0); and (iii) three models with two to four parameters fixed to zero (e.g., *λ*_S_ = 0, *μ*_O_ = 0) (see Table 4). Together, this approach allowed us to explicitly test the hypotheses that (i) speciation and extinction rates were different between outcrossing and selfing species; (ii) there were asymmetrical transition rates between mating types (i.e., test for irreversible [selfing to outcrossing] evolution; [37]); and (iii) each parameter was significantly different from zero.

**Table 4:** Median maximum likelihood (ML) estimates of parameters, log-likelihoods and AIC values of alternative BiSSE models estimated across 1,000 Beast trees of *Bulbophyllum* clade C. Speciation rates are *λ*, extinction rates are *μ*, and character transition rates are *q*. Outcrossing is coded as ‘O’ and selfing as ‘S’

Model	d.f.	*λ*_O_	*λ*_S_	*μ*_O_	*μ*_S_	*q*_OS_	*q*_SO_	Ln L	AIC	ΔAIC	% trees^1^ rejecting H_0_
*q*_OS_ = 0	5	0.59	0.85	1.05	6.41E-09	0	0.76	-66.97	143.94	-21.83	100
*λ*_O_ = 0	5	0	1.54	1.32E-05	5.88E-08	2.92	5.82	-63.04	136.09	-13.98	98.5
*λ*_O_ = *λ*_S_	5	0.66	0.66	1.26E-07	0.96	0.35	3.41E-06	-61.89	133.77	-11.67	99.3
Full ML	6	0.72	4.81E-07	5.21E-07	0.14	0.29	0.04	-58.68	129.35	-7.25	-
*q*_OS_ = *q*_SO_	5	0.72	1.25E-06	3.68E-06	1.03E-05	0.30	0.30	-58.86	127.73	-5.62	0
*q*_SO_ = 0	5	0.71	4.54E-07	8.25E-07	0.25	0.28	0	-58.80	127.60	-5.49	0
*μ*_O_ = *μ*_S_	5	0.71	2.15E-07	1.97E-06	1.97E-06	0.27	0.16	-58.75	127.50	-5.39	0
*μ*_S_ = 0	5	0.71	4.13E-07	6.50E-07	0	0.27	0.17	-58.75	127.50	-5.39	0
*μ*_O_ = 0	5	0.72	4.51E-08	0	0.14	0.29	0.04	-58.68	127.36	-5.25	0
*λ*_S_ = 0	5	0.72	0	1.32E-07	0.13	0.29	0.04	-58.68	127.35	-5.25	0
*λ*_S_ = 0, *μ*_O_ = 0	4	0.72	0	0	0.14	0.29	0.04	-58.68	125.35	-3.25	0
*λ*_S_ = 0, *μ*_O_ = 0, *q*_SO_ = 0	3	0.71	0	0	0.25	0.28	0	-58.80	123.60	-1.49	0
***λ***_**S**_ **= 0,** ***μ***_**O**_ **= 0,** ***μ***_**S**_ **= 0,** ***q***_**SO**_ **= 0**	**2**	**0.68**	**0**	**0**	**0**	**0.21**	**0**	**-59.05**	**122.11**	**0**	**0**

For each model a “=” sign indicates constrained model parameters, while all other parameters were allowed to freely vary. D.f. = degrees of freedom, Ln L = log-likelihood, AIC = Akaike Information Criterion, ΔAIC = change in AIC relative to the best model. Models are sorted in ascending order of ΔAIC values. The best fitting model based on the AIC is in bold

*The final column shows the percentage of trees where the more complex (= full) model provided a significant improvement (*P* < 0.05) over the simpler constrained model (H_0_) according to a likelihood-ratio test, where *P*-values were based on a *χ* ^2^ distribution with degrees of freedom (d.f.) equal to the difference in the number of free parameters allowed by the two models (i.e., alternative [= full model] versus null [= constrained model] hypotheses)

All models were fitted by ML nonlinear optimization across a sample of 1,000 of our Beast trees, using a heuristic starting point based (by default) on the state-independent BD model. For each model, ML parameter estimates, log-likelihoods and AIC values were recorded as median values (across the 1,000 trees), with the frequency distribution of full-model parameter values plotted along with their 95 % credible intervals. Model results were evaluated and compared using both median AIC values as well as likelihood ratio tests (LRTs). When differing by less than two ΔAIC units, models were considered being of essentially equivalent fit [87]. For each tree, LRTs were calculated as twice the difference in log-likelihoods between the full (more complex) model and the constrained (simpler) model (H_0_), which should follow a *χ*^2^ distribution with degrees of freedom equal to the difference in the number of free parameters allowed by the two models [40]. We then recorded the percentage of trees where the full model provided a significant improvement (*P* < 0.05) over the constrained model.

## Results

### Phylogenetics and hypothesis testing of monophyletic selfers

Our final dataset consisted of 670 bp from ITS, 765 bp of *PEPC*, 358 bp of *PI*, and 4712 bp of five plastid regions (*atp*I*–atp*H, *psb*A*–trn*H, *trn*D–*trn*E, *trn*T*–trn*S, *ycf1*). The combined nuclear/plastid dataset included 6,505 nucleotide positions, 477 of which were parsimony informative. The BI analysis of the four individual partitions [Additional files 3, 4, 5 and 6] yielded largely congruent tree topologies albeit with different levels of resolution. In our focal BI tree of the combined analysis (Fig. 2), which produced highest node support values, members of clade C grouped according to their sectional affiliation (all PP = 1; BP ≥ 99). The species-poor sect. *Bifalcula* and the monotypic sect. *Humblotiorchis* (comprising one selfing species each) formed successive sister taxa to the species-rich sect. *Calamaria* (including six selfers). While these sectional groupings and relationships are in agreement with previous phylogenetic studies [50], our increased taxon and gene sampling resulted in better-resolved interspecies relationships, with most intra-specific accessions recovered as monophyletic (PP > 0.95; BP 55–100), except for the paraphyletic *B. implexum* and the likely polyphyletic *B*. sp. nov. ‘*E1*’. However, especially within sect. *Calamaria*, several internal branches were not supported well (PP < 0.83; BP ≤ 50), indicating some phylogenetic uncertainty for character state reconstructions. Nonetheless, the scattered phylogenetic distribution of the eight selfing species strongly suggested that they do not form a monophyletic group (Fig. 2). In support of this, tree topology tests (Table 2) decisively rejected models in which all selfing species of clade C, or only those of its sublineages (sects. *Calamaria + Humblotiorchis*; or sect. *Calamaria*) were constrained to be monophyletic (all BF values > 10), pointing at multiple independent shifts in mating type across clade C (but see below).

### Evolutionary transitions of mating type in clade C

Our Mk2 (MCMC) ancestral state reconstructions of mating type over 5,000 BI trees yielded mostly similar results when compared to those obtained with ML reconstructions over 1,000 Beast trees following the best fitting BiSSE model (Fig. 3a, b; Table 4). Both analyses identified outcrossing as the most probable ancestral state of clade C, and revealed that transitions to selfing were in general concentrated in terminal branches (marked with asterisks in Fig. 3a, b). However, under the Mk2 reconstruction, two subterminal nodes had relatively high probabilities of being selfing (i.e., the ancestral nodes of, respectively, *B. occultum*/*B. bicoloratum*/*B*. sp. nov. ‘*D*’, and *B. pusillum*/*B.quadrifarium*; Fig. 3a), resulting in six transitions. By contrast, the best fitting BiSSE model reconstructed all internal nodes as being outcrossing, resulting in eight independent transitions to selfing along terminal branches leading to extant species (Fig. 3b). In any event, these results qualitatively indicate that mating type transitions within clade C are a strikingly recurrent evolutionary phenomenon, but which requires further temporal delimitation and quantitative evidence as provided in the following sections.

### Diversification times

Our relaxed molecular clock analyses (Fig. 3c, [Additional file 8]) dated the onset of diversification (crown age) of clade C to the Late Tertiary/Early Pliocene, at ca. 5.3 Ma (95 % highest posterior density [HPD] of 8.5–2.7 Ma). By contrast, the most recent common ancestor (MRCA) of sects. *Humblotiorchis/Calamaria* was placed in the mid-Pliocene, at ca. 4.4 Ma (95 % HPD of 5.3–3.4 Ma), and the crown age of sect. *Bifalcula* in the Late Pliocene, at ca. 3.1 Ma (95 % HPD of 4.2–2.0 Ma). The origins of most extant clade C species were dated to the Quaternary (≤ 2.6 Ma), excepting few species inferred as Late Tertiary/Pliocene relics (i.e., *B. erectum*, *B. humblotii*, *B. capuronii*). Notably, five of the eight selfing species originated within a relatively recent time frame starting at about the mid-Pleistocene (ca. 1.3 Ma; see node marked by an asterisk in Fig. 3c; 95 % HPD of 1.9–0.8 Ma). By contrast, of the three remaining selfers, one originated in the Early Pleistocene (*B. complanatum* [ca. 2.4 Ma]) and two in the Pliocence (*B. erectum* [ca. 3.1 Ma] and *B. humblotii* [ca. 4.37 Ma]; Fig. 3c). All these latter taxa evolved selfing independently (Fig. 3), leaving the possibility of a more complex history of selfing (in terms of speciation, extinction and/or transitions) over extended time scales.

### Diversification and transition rates

The LTT plots for the phylogeny of clade C (inset Fig. 3c), derived from the chronogram (Fig. 3c) and 1,000 dated Beast trees, suggested an almost constant lineage accumulation over time, with no apparent upturn near the present as typical for either high (background) extinction rates or recent and rapid speciation rates [82, 85]. Concomitantly, the ∆AIC_RC_ test in Laser (Table 3) indicated no significant departure of the best-fit rate-constant model, i.e., pure-birth (PB) without extinction (AIC = -48.97), from the best-fit rate-variable model (YR3) with likewise no extinction but three distinct rates of diversification (AIC = -52.22). The overall speciation rate (*λ*) of clade C calculated under the null (PB) model was 0.53 species myr^-1^. This value is within the range observed for other plant taxa that have been identified as undergoing rapid rates of relatively recent speciation (e.g., [27, 88, 89]).

**Fig. 4:**
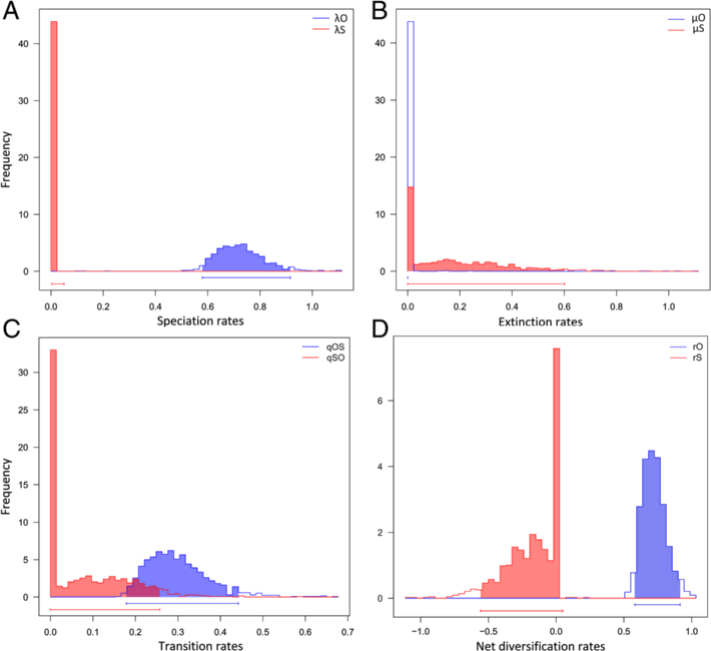
Frequency distributions of diversification and transition rates. **a** Speciation rates, (**b**) extinction rates, (**c**) character transition rates, and (**d**) net diversification rates for outcrossing (subscript ‘O’) and selfing (‘S’) lineages estimated under the full (six-parameter) BiSSE model across 1,000 dated Beast trees using maximum likelihood procedures. The 95 % credible intervals of parameter values are shown along the x-axes

Although the above results indicate no shifts in lineage diversification over time within clade C, mating system *per se* might have conferred unequal probabilities of speciation and extinction [14, 33]. In order to address this issue, we calculated mating-type-dependent speciation and extinction rates along with transition rates (in units myr^-1^) under the full (six-parameter) BiSSE model (and constrained variants thereof) across 1,000 dated Beast trees using ML procedures (Table 4; Fig. 4). According to the full model, speciation rates were high in outcrossing lineages (*λ*_O_ = 0.72) but next to zero in selfing lineages (*λ*_S_ = 4.81E^-07^), whereas the reverse was true for extinction rates (*μ*_O_ = 5.21E^-07^ vs. *μ*_S_ = 0.14). As a result, net diversification rates were negative in selfers (*r*_S_ = -0.14) but still high in outcrossers (*r*_O_ = 0.72). Rates of transition from outcrossing to selfing were high (*q*_OS_ = 0.29) but near-zero for the reverse (*q*_SO_ = 0.04). For speciation and net diversification rates, the corresponding frequency distributions of parameter values showed no overlap in their 95 % credible intervals (indicating great certainty in these contrasting estimates), but overlap did occur for extinction rates, and more unexpectedly, transition rates (Fig. 4). When we compared the fit of the full (complex) model against each of twelve simpler constrained (H_0_) models (Table 4), likelihood-ratio tests (LRTs) clearly rejected models with zero outcrosser speciation (*λ*_O_ = 0), equal speciation (*λ*_O_ = *λ*_S_), and zero transition from outcrossing to selfing (*q*_OS_ = 0). By the same tests, all other constrained models provided no better fit than the full model. However, using median AIC values, the best-fit model overall (marked bold in Table 4) was one in which the speciation rate of selfers, and the extinction rates of both outcrossers and selfers, were set to zero, along with irreversible transitions from outcrossing to selfing (i.e., *λ*_S_ = 0, *μ*_O_ = 0, *μ*_S_ = 0, *q*_SO_ = 0). The resulting estimates of this two-parameter model were *λ*_O_ = 0.68 and *q*_OS_ = 0.21. Significantly though, a three-parameter model, in which the extinction rate of selfers (*μ*_S_) was allowed to vary (i.e., *λ*_S_ = 0, *μ*_O_ = 0, *q*_SO_ = 0), was only marginally less supported (ΔAIC = -1.49) and suggested a moderate extinction rate for selfing lineages (*μ*_S_ = 0.25); the other estimates of this ‘second-best model’ were comparable to those of the best-fit model (*λ*_O_ = 0.71, *q*_OS_ = 0.28) (Table 4). Following our best-fit model, all the internal nodes of the 1,000 Beast trees were reconstructed to be outcrossing (Fig. 3b, c), and the same applied to the second best model (not shown).

## Discussion

According to recent reformulations [7, 14], Stebbins’ SEDE hypothesis conflates two distinct claims: (1) transition rates from selfing to outcrossing are zero; and (2) the net diversification rate (speciation rate minus extinction rate) is negative for selfing taxa, implying a higher risk of extinction (i.e., shorter evolutionary lifespan) for selfing relative to outcrossing taxa [2, 17, 20, 25]. Our results for Madagascan *Bulbophyllum* clade C support the first claim of the SEDE hypothesis (irreversibility of transitions to selfing) and we find some, albeit no definite evidence for the second (higher extinction rates for selfers). In principle, therefore, our data are largely congruent with predictions raised by the SEDE hypothesis [14], but some of the patterns observed suggest that selfing represents a starting-point rather than a terminus of mating-system evolution in these tropical orchids. Below we discuss the evidence and possible causes for the strikingly recurrent and probably very recent transitions to selfing in our study system.

### Multiple origins and irreversibility of selfing

We support the first assumption of the SEDE hypothesis in that outcrossing (rostellum presence, Type I) represents the ancestral state of *Bulbophyllum* clade C (Fig. 3) and that transitions exclusively occurred from outcrossing to selfing (Types II/III), as also confirmed by the state-dependent BiSSE analyses (Table 4). Although our ancestral state reconstructions using the Mk2 model indicate two subterminal nodes where transitions could have occurred (Fig. 3a), this is not supported by the ML reconstructions under the best fitting BiSSE model (Fig. 3b, c). Accordingly, selfing via rostellum loss (Type II) most likely evolved independently at eight (rather than only six) times within this clade of 30 species. In addition, selfing via rostellum receptivity (Type III) evolved uniquely in the trimorphic *B. bicoloratum* [47, 48]. These results also imply that the sharing of selfing/outcrossing morph variation among the eight focal species is unlikely due to the retention of an ancestral polymorphism, and the consistent rejection of their monophyly (Table 2) further supports this view.

Unidirectional transitions from outcrossing to selfing are commonly reported in flowering plants [3, 18–20, 24, 26, 28] but see [25, 27]. However, this is one of few studies to estimate a time-calibrated transition rate to selfing based on a discrete-state character. The only comparable studies we are aware of, focussing on the breakdown of SI in Solanaceae [39] and the loss of heterostyly in *Primula* [56], reported higher and lower rates than observed here (i.e., *q*_OS_ 0.56 and 0.04–0.10, respectively, vs. 0.21 [this study], all in units myr^-1^). We note that BiSSE is currently the best method to test character state irreversibility, as it is less prone to falsely infer reversions [7, 14], and has been used previously for clades of similarly small size (e.g., [37, 78, 90–93]). However, it has relatively low power to detect rate differences among states of a binary trait when dealing with topologies of less than about 300 terminals and a high degree of asymmetry in tip states [33–35]. Our clade C phylogeny has 30 tips of which 8 (26.7 %) have state one (i.e., selfing). The results of our BiSSE analysis must therefore be treated cautiously (see also below).

The fact that selfing has arisen multiple, independent times within clade C may imply a selective advantage of this trait ([55, 92, 94]; see further below), and that inbreeding depression, as the principal counterweight to this advantage (e.g., [13, 14]), has not impeded the evolution of selfing in these tropical orchids. In support of the latter hypothesis, controlled mating experiments have found no evidence of inbreeding depression at the level of seed viability and fruit set in, respectively, the outcrossing morph of Madagascan *B. occultum* (U. Jaros, unpubl. data) and neotropical *Bulbophyllum* species [95]. There is also little evidence of such early-acting (pre-dispersal) inbreeding depression in other self-compatible orchid species [96–98]. At least in part, this may be explained by the fact that selfed offspring usually does not depend on parental (i.e., endosperm) resources in Orchidaceae [43, 99].

But what intrinsic factor(s) then could promote the parallel evolution of selfing via rostellum loss within clade C? One possible genetic explanation for this parallelism is that it ultimately reflects recurrent, perhaps loss-of-function mutations at only one or a few genes affecting the timing of rostellum development (see also [48]). Morphological divergence within and between plant species involving the presence versus absence of structures, as observed here for rostellum type, has often been demonstrated to have such a simple genetic basis [100–103], and selection on such traits should lead to rapid evolutionary response [101, 103, 104]. On the other hand, given the morphological and developmental complexity of the rostellum [47, 48, 105], it is not unexpected that we find no evidence for the regain of this organ. In general, such kind of irreversibility is expected from ‘Dollo’s Law’, which posits that complex characters, once lost, are unlikely to re-evolve [2, 106], as also demonstrated for other morphological structures associated with outcrossing (e.g., heterostyly: [2, 20] but see [107]). However, at least for the orchid species studied here, a non-mutually exclusive explanation is that transitions to selfing evolved too recently to detect reversals (see below).

### Explanations for the ‘twiggy’ phylogenetic distribution of selfing

It has long been noted that selfing taxa often occur in scattered positions at the tips of plant phylogenies (e.g., *Amsinckia*: [20]; Triticeae: [21]; *Leavenworthia*: [22, 23]. Such’twiggy’ (or ‘tippy’) distributions have typically been interpreted as a hallmark signature of the elevated extinction risk of selfers, as proposed by the second part of the SEDE hypothesis [15, 17]. However, these’twiggy’ distributions can also arise if transitions from the ancestral state are rare or asymmetrical, but this has rarely been tested using sophisticated analysis methods, such as the state-dependent BiSSE model [40, 41, 108, 109], and with partly contrasting results. For example, in *Primula* [56], this latter model proved useful in explaining the phylogenetic ‘twigginess’ of the selfing state by highly asymmetrical transitions to selfing from outcrossing, albeit coupled with *increased* speciation and *zero* extinction (positive *r*). Interestingly, these results demonstrate that selfing lineages, perhaps freed from selective constraints, can evolve at higher rates than outcrossing ones, but also suggests that sufficient time is needed for the negative effects of selfing to accumulate ([15]; J. de Vos, pers. comm.).

For *Bulbophyllum*, our data best fit two BiSSE models in which the outcrossing state is generally associated with high rates of speciation and zero extinction (*λ*_O_ = 0.68/071; *μ*_O_ = 0), and the selfing state with zero speciation (*λ*_S_ = 0) (Table 4); however, we did not find definite evidence for the prediction of increased extinction of selfing species, even though the second-best model with moderate extinction (*μ*_S_ = 0.25; and thus negative *r*) fit reasonably well compared to the best-fit model without extinction (*μ*_S_ = 0) (ΔAIC < 2; Table 4). This lack of ‘measurable’ extinction may simply reflect the above caveat about the statistical power of BiSSE, coupled with the relatively short branch lengths subtending the selfing species of *Bulbophyllum* (Fig. 3c). In sum, the results of our BiSSE analyses must be regarded as inconclusive on whether or not extinction contributes to the twiggy phylogenetic distribution of selfing in clade C (Fig. 3), and thus in addition to the repeated (and irreversible) transition to this character, coupled with strongly reduced (zero) speciation in selfing compared to outcrossing taxa. That said, if selfing had evolved only recently in this group, as discussed below, then time might have been insufficient to detect elevated extinction and/or speciation rates associated with this state ([40, 56]; see also [92]).

### Relatively recent shifts in mating system

There is mounting evidence from population genetic studies indicating that outcrossing-to-selfing transitions are often rather recent in flowering plants [7, 14], with most origins dated to Quaternary (≤ 2.6 Ma) or even millennial time scales [22, 110–﻿115]. Our results lend support to this view in that selfing evolved independently, in all probability at five times, within clade C since about the mid-Pleistocene (ca. ≤ 1.3 Ma; see node marked by an asterisk in Fig. 3c). However, given that all eight transitions are most likely associated with terminal branches leading to extant species (Fig. 3c), it remains unclear when selfing evolved within a particular species. This could have happened at any point along such a branch, with the possibility in three species (i.e., *B. complanatum*, *B. erectum*, *B. humblotii*) of tracing back to even Early Pleistocene/Pliocene times. Nonetheless, for each of the eight focal species, there is circumstantial evidence to suggest that selfing evolved only recently, possibly over Late Quaternary, if not historic time scales.

First, molecular genetic data in particular from Brassicaceae indicate that selfing tends to evolve much more recently within a species than the time of separation from its closest extant relative [13, 113]. For example, the common ancestor of *Arabidopsis thaliana* and *A. lyrata* (which likely diverged ca. 5 Ma [113] or 8.0–17.9 Ma [114]) was presumably outcrossing and self-incompatible, yet *A. thaliana* is thought to have acquired self-compatibility only within the last 1.8 million years [113–115]. An even more recent origin of selfing seems plausible for each of the eight *Bulbophyllum* species, where – unlike in *A. thaliana* – selfing individuals still co-exist with ancestrally outcrossing ones at the intraspecific level. In addition, apart from their diagnostic rostellum traits, these conspecific morphs hardly differ in other morphological or functional changes to the flowers [47, 48]. This further suggests that these morphs only recently diverged, whereby insufficient time may have elapsed for such a ‘floral selfing syndrome’ [116] to develop, as has been demonstrated in a large number of genera where selfing lineages have been derived from outcrossing ancestors (reviewed in [117]; but see [118]). On the other hand, if selfing had evolved in the more distant past in *Bulbophyllum*, we would expect to observe at least some of the older species (e.g., *B. humblotii*) to be entirely fixed for selfing, especially given the evidence for low inbreeding depression in this group (see above). Accordingly, the eight clade C species that currently vary for mating type may well be in a transitory phase towards increased selfing, as postulated for other species polymorphic for selfing/outcrossing individuals [55, 112].

### Potential environmental catalysts for the evolution of selfing

Observations on cultivated material suggest that the selfing morphs (Types II/III) of Madagascan *Bulbophyllum* species are unlikely to reproduce through outcrossed pollinia, and thus most likely behave as exclusive rather than partial selfers [48]. If this holds true in nature, then the origin and maintenance of selfing within clade C is unlikely due to a mere ‘selective transmission advantage’ of selfing [5–7], because this advantage disappears when pollen discounting (i.e., a reduction in male outcross success that results from selfing) is maximised [7]. It is feasible, therefore, that recurrent transitions to selfing within this clade mostly, if not exclusively, reflect an adaptive ‘reproductive assurance strategy’ [8–10] in response to similar selective environments that favour reproduction when pollinators or mates are rare or absent, conditions that might accrue from island colonization or habitat fragmentation [7, 119, 120]. In our system, however, we find no support for the oft-invoked idea that the evolution of selfing promotes long-distance colonization [2, 8, 15, 121]. Although in some species (*B. humblotii*, *B. occultum*, *B. pusillium*) selfing may have increased colonizing ability of neighbouring islands, such as La Réunion, all these taxa vary for mating type in Madagascar [48, 54]. On the other hand, we suspect that potential catalysts that may have initiated fragmented habitat conditions in Madagascar include (1) climate-induced vegetation shifts during the (Late) Quaternary [122–126]; and (2) human-mediated degradation of primary forest over the last 1,800 years [127–129]. Effects of both Quaternary climate change and human-modified landscapes on the evolution of selfing in flowering plants are well documented [22, 104, 111, 114, 130]. Our phylogenetic data cannot determine the relative importance of these ‘shallow-time’ effects to mating system evolution in *Bulbophyllum* clade C; however, they emphasize the need for comprehensive field and population-level data (e.g., on morph frequencies, pollinators, demography, niche requirements, phylogeography, and genomics) to elucidate the past and/or present selective environments under which selfing evolved within a particular *Bulbophyllum* species [131].

## Conclusions

In this study, we examined the evolution of selfing (auto-pollination) in Madagascan *Bulbophylum* orchids (clade C) in the light of two predictions raised by the classical SEDE hypothesis: (1) irreversible transitions from outcrossing to selfing; and (2) elevated extinction rates associated with the selfing state. Although we find convincing support for the first prediction, results remain inconclusive on whether or not extinction contributes to the twiggy phylogenetic distribution of selfing in clade C. Despite this latter limitation, we suspect that selfing has evolved too recently in this group to detect elevated extinction and/or speciation rates associated with this state. We further hypothesize that a simple genetic basis of rostellum loss may explain the strikingly recurrent transitions to selfing in our study system (i.e., exclusively *within* species), perhaps reflecting rapid response to parallel and novel selective environments over Late Quaternary (≤ 1.3 Ma) time scales. Future demographic and genomic studies of these and other tropical orchids with an apparent lability towards rostellum abortion have thus the potential to unravel novel perspectives on the ability of some natural systems to rapidly evolve selfing as a means of avoiding extinction in the face of ever changing environments.

## Availability of supporting data

The data sets supporting the results of this article are included within the article (and its Additional files 1, 2, 3, 4, 5, 6, 7, 8, 9, 10, 11, 12 and 13). Nucleotide sequence data supporting the results of this article are available in GenBank [see Additional file 1]. The phylogenetic data sets supporting the results of this article are available in the Dryad Digital Repository [doi:10.5061/dryad.35935].

## Additional files

Additional file 1:
**Summary of specimens used for the phylogenetic study of Madagascan** ***Bulbophyllum*** **clade C.** Including information on species distribution, geographic origin of specimens (as far as known), voucher numbers, and associated GenBank (NCBI) sequence accession numbers for the three nuclear regions (nrITS, *PEPC*, *pistillata/globosa* [*PI*]) and the five plastid regions (*atpI–atpH*, *psbA–trnH*, *trnD–trnE*, *trnT–trnS*, *yfc1*). (DOCX 43 kb) Additional file 2:
**Newly designed primers for** ***PEPC*** **and** ***pistillata*** **/** ***globosa*** **(** ***PI*** **)** **based on the studies of [63] and [64].** (DOCX 13 kb) Additional file 3:
**Majority-rule consensus tree of Madagascan** ***Bulbophyllum*** **clade C from the Bayesian analysis of the plastid five-gene** **(** ***atp*** **I** ***–atp*** **H,** ***psb*** **A** ***–trn*** **H,** ***trn*** **D–** ***trn*** **E,** ***trn*** ***–trn*** **S,** ***ycf1*** **) dataset.** Closed circles indicate nodes with Bayesian posterior probability (PP) of 1 and parsimony bootstrap percentage (BP) ≥ 85. Branches only weakly supported by Bayesian analysis (PP ≤ 0.95) are indicated through stippled lines. (TIFF 1199 kb) Additional file 4:
**Majority-rule consensus tree of Madagascan** ***Bulbophyllum*** **clade C from the Bayesian analysis of the nrITS dataset.** Closed circles indicate nodes with PP of 1 and BP ≥ 85. Branches only weakly supported by Bayesian analysis (PP ≤ 0.95) are indicated through stippled lines. (TIFF 1102 kb) Additional file 5:
**Majority-rule consensus tree of Madagascan** ***Bulbophyllum*** **clade C from the Bayesian analysis of the single/low copy nuclear marker** ***PEPC.*** Closed circles indicate nodes with PP of 1 and BP ≥ 85. Branches only weakly supported by Bayesian analysis (PP ≤ 0.95) are indicated through stippled lines. (TIFF 560 kb) Additional file 6:
**Majority-rule consensus tree of Madagascan** ***Bulbophyllum*** **clade C from the Bayesian analysis of the single/low copy nuclear marker** ***PI.*** Closed circles indicate nodes with PP of 1 and BP ≥ 85. Branches only weakly supported by Bayesian analysis (PP ≤ 0.95) are indicated through stippled lines. (TIFF 1626 kb) Additional file 7:
**Estimating times of divergence: Secondary calibration approach.** (DOCX 25 kb) Additional file 8:
**Secondarily calibrated chronogram of the genus** ***Bulbophyllum*** **used for the estimation of the crown group age of Madagascan** ***Bulbophyllum*** **clade C.** Branches only weakly supported by Bayesian analysis (PP < 0.90) are indicated through stippled lines. Ages of major nodes are indicated by yellow circles, and the corresponding age estimates can be found in [Additional file 13]. Colored circles indicate age-constrained nodes (red circle, root node; cyan circles, biogeographic [island age] calibration points). (TIFF 5850 kb) Additional file 9:
**Time-calibrated phylogeny of the Orchidaceae from the** **Beast** **analysis of the plastid two-gene** **(** ***mat*** **K,** ***rbc*** **L) dataset, used for the estimation of the stem group age of** ***Bulbophyllum.*** Numbers at nodes are median ages in million years ago (Ma) (see also [Additional file 12]). Branches only weakly supported by Bayesian analysis (PP < 0.90) are indicated through stippled lines. Colored circles indicate age-constrained nodes (red circle, root node; green circles, fossil-based calibration points). (TIFF 1191 kb) Additional file 10:
**GenBank (NCBI) accession numbers of plastid DNA (** ***mat*** **K,** ***rbc*** **L) sequences of Orchidaceae used to estimate the stem node age of the genus** ***Bulbophyllum.*** (DOCX 20 kb) Additional file 11:
**List of 277 accessions of nrDNA (ITS) sequences of** ***Bulbophyllum*** **and** ***Dendrobium*** **used to estimate the crown node age of Madagascan** ***Bulbophyllum*** **clade C.** (DOCX 74 kb) Additional file 12:
**Age estimates of the family Orchidaceae and various subclades as compared to previous ones.** (DOCX 17 kb) Additional file 13:
**Infrageneric age estimates of the genus** ***Bulbophyllum.*** (DOCX 17 kb)
