# Supplementary material for: Multiple independent origins of auto-pollination in tropical orchids (Bulbophyllum) in light of the hypothesis of selfing as an evolutionary dead end
Source: BMC Evol Biol. 2015 Sep 16;15:192. doi: 10.1186/s12862-015-0471-5 (PMC4574068; doi:10.1186/s12862-015-0471-5)
Supplement: Additional file 1: — Summary of specimens used for the phylogenetic study of Madagascan Bulbophyllum clade C. Including information on species distribution, geographic origin of specimens (as far as known), voucher numbers, and associated GenBank (NCBI) sequence accession numbers for the three nuclear regions (nrITS, PEPC, pistillata/globosa [PI]) and the five plastid regions (atpI–atpH, psbA–trnH, trnD–trnE, trnT–trnS, yfc1). (DOCX 43 kb) [file 12862_2015_471_MOESM1_ESM.docx]

**Additional file 1**

**Gamisch et al. “Multiple independent de novo origins of auto-pollination in tropical orchids (*Bulbophyllum*) in light of the hypothesis of selfing as an evolutionary dead end”**

**Additional file 5: Summary of specimens used for the phylogenetic study of Madagascan *Bulbophyllum* clade C.** Including information on species distribution, geographic origin of specimens (as far as known), voucher numbers, and associated GenBank (NCBI) sequence accession numbers for the three nuclear regions (nrITS, *PEPC*, *pistillata*/*globosa* [*PI*]) and the five plastid regions (*atp*I*–atp*H, *psb*A*–trn*H, *trn*D–*trn*E, *trn*T*–trn*S, *yfc1*).

| Section*/*species | | | Distribution/  Origin* | Voucher number ^†^ | GenBank accession number | | | | | | | |
| --- | --- | --- | --- | --- | --- | --- | --- | --- | --- | --- | --- | --- |
|  |  |  |  |  | nrITS | *PEPC* | *pistillata*  */globosa* | *atp*I*–atp*H | *psb*A*–trn*H | *trn*D–*trn*E | *trn*T*–trn*S | *ycf1* |
| *Bifalcula* Schltr. | | |  |  |  |  |  |  |  |  |  |  |
|  | *B. capuronii* Bosser | | MAD | FS1010 (SZU) | EF195966 | KJ558625 | KJ558773 | KJ558989 | EF200383 | KJ558661 | KJ558889 | KJ558559 |
|  | *B. capuronii* | |  | FS5484 (SZU) | KJ558736 | KJ558622 | KJ558803 | KJ558992 | KJ558926 | KJ558683 | KJ558860 | KJ558562 |
|  | *B. complanatum* H.Perrier | | MAD | FS1298 (SZU) | EF633602 | KJ558649 | KJ558785 | KJ558964 | KJ558910 | KJ558667 | KJ558877 | KJ558534 |
|  | *B. complanatum* | |  | FS5762 (SZU) | KJ558748 | KJ558620 | KJ558815 | KJ558994 | KJ558938 | KJ558695 | KJ558848 | KJ558564 |
|  | *B. complanatum* | |  | FS5866 (SZU) | KJ558750 | KJ558613 | KJ558817 | KJ559002 | KJ558940 | KJ558697 | KJ558846 | KJ558572 |
|  | *B. implexum* Jum. & H.Perrier | | MAD | FS1207 (SZU) | EF196031 | KJ558651 | KJ558781 | KJ558960 | EF200385 | EF202199 | KJ558881 | KJ558531 |
|  | *B. implexum* | |  | FS6042 (SZU) | KJ558757 | KJ558618 | KJ558825 | KJ558996 | KJ558948 | KJ558705 | KJ558838 | KJ558566 |
|  | *B. minutum* Thouars | | MAD | FS1006 (SZU) | EF196061 | KJ558628 | KJ558771 | KJ558986 | EF200386 | EF202198 | KJ558891 | KJ558556 |
|  | *B. minutum* | |  | FS5306 (SZU) | KJ558732 | KJ558615 | KJ558799 | KJ559000 | KJ558922 | KJ558679 | KJ558864 | KJ558570 |
|  | *B.* sp. nov. *‘A’* | | MAD | FS5921 (SZU) | - | KJ558612 | KJ558820 | KJ559003 | KJ558943 | KJ558700 | KJ558843 | KJ558573 |
|  | *B.* sp. nov. *‘A’* | | MAD | FS2707 (SZU) | KJ558722 | KJ558646 | KJ558789 | KJ558968 | KJ558912 | KJ558669 | KJ558874 | KJ558538 |
|  | *B.* sp. nov. *‘B’* | | MAD | FS5534 (SZU) | KJ558740 | KJ558644 | KJ558807 | KJ558970 | KJ558930 | KJ558687 | KJ558856 | KJ558540 |
| *Calamaria* Schltr. | | |  |  |  |  |  |  |  |  |  |  |
|  | *B. bicoloratum* Schltr. | | MAD | FS1052 (SZU) | EF195964 | KJ558634 | KJ558827 | KJ558980 | EF200387 | EF202200 | KJ558836 | KJ558550 |
|  | *B. bicoloratum* | |  | FS807 (SZU) | KJ558714 | KJ558637 | KJ558766 | KJ558977 | KJ558903 | KJ558659 | KJ558896 | KJ558547 |
|  | *B. cirrhoglossum* H.Perrier | | MAD | FS4125 (SZU) | KJ558728 | KJ558643 | KJ558795 | KJ558971 | KJ558918 | KJ558674 | KJ558868 | KJ558541 |
|  | *B. elliotii* Rolfe | | MAD | FS863 (SZU) | EF195976 | KJ558632 | KJ558770 | KJ558982 | EF200388 | EF202202 | KJ558892 | KJ558552 |
|  | *B. elliotii* | |  | FS4137 (SZU) | KJ558729 | KJ558642 | KJ558796 | KJ558972 | KJ558919 | KJ558676 | KJ558867 | KJ558542 |
|  | *B. erectum* Thouars | | MAD | FS1022 (SZU) | KJ558716 | KJ558629 | KJ558774 | KJ558985 | KJ558905 | KJ558662 | KJ558888 | KJ558555 |
|  | *B. erectum* | |  | FS1023 (SZU) | KJ558717 | KJ558635 | KJ558775 | KJ558979 | KJ558906 | KJ558663 | KJ558887 | KJ558549 |
|  | *B. hildebrandtii* Rchb.f. | | MAD | FS1133 (SZU) | EF195983 | KJ558654 | KJ558777 | KJ558956 | EF200392 | EF202206 | KJ558885 | KJ558527 |
|  | *B. hildebrandtii* | |  | FS5648 (SZU) | KJ558743 | KJ558619 | KJ558810 | KJ558995 | KJ558933 | KJ558690 | KJ558853 | KJ558565 |
|  | *B. hildebrandtii* | |  | FS5682 (SZU) | KJ558744 | KJ558602 | KJ558811 | KJ559013 | KJ558934 | KJ558691 | KJ558852 | KJ558583 |
|  | *B. hildebrandtii* | |  | FS1169 (SZU) | KJ558720 | KJ558653 | KJ558779 | KJ558958 | KJ558909 | KJ558666 | KJ558883 | KJ558529 |
|  | *B. histrionicum* G.A.Fischer & P.J.Cribb | | MAD | FS1232 (SZU) | EF196062 | - | KJ558783 | KJ558962 | EF200397 | EF202211 | KJ558879 | KJ558533 |
|  | *B. histrionicum* | |  | FS6058 (SZU) | KJ558758 | KJ558603 | KJ558826 | KJ559012 | KJ558949 | KJ558706 | KJ558837 | KJ558582 |
|  | *B. histrionicum* | |  | FS6018 (SZU) | KJ558753 | KJ558605 | KJ558821 | KJ559010 | KJ558944 | KJ558701 | KJ558842 | KJ558580 |
|  | *B. lecouflei* Bosser | | MAD | FS2715 (SZU) | KJ558723 | KJ558645 | KJ558790 | KJ558969 | KJ558913 | KJ558670 | KJ558873 | KJ558539 |
|  | *B. lecouflei* | |  | FS1278 (SZU) | EF196029 | - | KJ558784 | KJ558963 | EF200393 | EF202207 | KJ558878 | - |
|  | *B. luteobracteatum* Jum. & H.Perrier | | MAD | FS5512_OR1535 (SZU) | KJ558738 | KJ558595 | KJ558805 | KJ559020 | KJ558928 | KJ558685 | KJ558858 | KJ558590 |
|  | *B. luteobracteatum* | |  | FS5512_OR1257 (SZU) | KJ558737 | KJ558611 | KJ558804 | KJ559004 | KJ558927 | KJ558684 | KJ558859 | KJ558574 |
|  | *B. obtusatum* (Jum. & H.Perrier) Schltr. | | MAD | FS1170 (SZU) | EF196039 | KJ558652 | KJ558780 | KJ558959 | EF200395 | EF202209 | KJ558882 | KJ558530 |
|  | *B. obtusatum* | |  | FS5736 (SZU) | KJ558747 | KJ558601 | KJ558814 | KJ559014 | KJ558937 | KJ558694 | KJ558849 | KJ558584 |
|  | *B. occultum* Thouars | | **MAD,** REU, MAU, COM | FS603 (SZU) | KJ558760 | KJ558624 | KJ558829 | KJ558990 | KJ558951 | KJ558708 | KJ558834 | KJ558560 |
|  | *B. occultum* | |  | FS3813 (SZU) | KJ558724 | KJ558636 | KJ558791 | KJ558978 | KJ558914 | KJ558671 | KJ558872 | KJ558548 |
|  | *B. occultum* | |  | FS5513 (SZU) | KJ558739 | KJ558614 | KJ558806 | KJ559001 | KJ558929 | KJ558686 | KJ558857 | KJ558571 |
|  | *B. pervillei* Rolfe | | MAD | FS818 (SZU) | EF196049 | KJ558633 | KJ558767 | KJ558981 | EF200400 | EF202214 | KJ558895 | KJ558551 |
|  | *B. pervillei* | |  | FS5444 (SZU) | KJ558734 | KJ558621 | KJ558801 | KJ558993 | KJ558924 | KJ558681 | KJ558862 | KJ558563 |
|  | *B. pusillum* var. *pusillum* (H.Perrier) G.A.Fischer & P.J.Cribb | | **MAD**, REU | FS1459 (SZU) | EF196057 | - | KJ558786 | KJ558965 | EF200398 | EF202212 | - | KJ558535 |
|  | *B. pusillum* var. *pusillum* | |  | FS4885 (SZU) | KJ558730 | KJ558597 | KJ558797 | KJ559018 | KJ558920 | KJ558677 | KJ558866 | KJ558588 |
|  | *B. quadrifarium* Rolfe | | MAD | FS826 (SZU) | EF196028 | KJ558626 | KJ558768 | KJ558988 | EF200396 | EF202210 | KJ558894 | KJ558558 |
|  | *B. quadrifarium* | |  | FS827 (SZU) | KJ558715 | KJ558631 | KJ558769 | KJ558983 | KJ558904 | KJ558660 | KJ558893 | KJ558553 |
|  | *B. quadrifarium* | |  | FS5445 (SZU) | KJ558735 | KJ558616 | KJ558802 | KJ558999 | KJ558925 | KJ558682 | KJ558861 | KJ558569 |
|  | *B. quadrifarium* | |  | FS6021 (SZU) | KJ558754 | KJ558607 | KJ558822 | KJ559008 | KJ558945 | KJ558702 | KJ558841 | KJ558578 |
|  | *B. rubrum* Jum. & H.Perrier | | MAD | FS5603_OR1259 (SZU) | KJ558741 | KJ558610 | KJ558808 | KJ559005 | KJ558931 | KJ558688 | KJ558855 | KJ558575 |
|  | *B. rubrum* | |  | FS5603_OR1268 (SZU) | KJ558742 | KJ558608 | KJ558809 | KJ559007 | KJ558932 | KJ558689 | KJ558854 | KJ558577 |
|  | *B. ruginosum* H.Perrier | | MAD | FS5885_OR1400 (SZU) | KJ558751 | KJ558598 | KJ558818 | KJ559017 | KJ558941 | KJ558698 | KJ558845 | KJ558587 |
|  | *B. ruginosum* | |  | FS5885_OR1519 (SZU) | KJ558752 | KJ558596 | KJ558819 | KJ559019 | KJ558942 | KJ558699 | KJ558844 | KJ558589 |
|  | *B. pusillum* var. *sambiranense* G.A.Fischer & P.J.Cribb | | MAD | FS2019 (SZU) | EF195973 | KJ558647 | KJ558788 | KJ558967 | EF200389 | EF202203 | KJ558875 | KJ558537 |
|  | *B. pusillum* var. *latibracteatum* (H.Perrier) G.A.Fischer & P.J.Cribb | | MAD | FS5718 (SZU) | KJ558745 | KJ558599 | KJ558812 | KJ559016 | KJ558935 | KJ558692 | KJ558851 | KJ558586 |
|  | *B. pusillum.* var. *latibracteatum* | | MAD | FS5719 (SZU) | KJ558746 | KJ558600 | KJ558813 | KJ559015 | KJ558936 | KJ558693 | KJ558850 | KJ558585 |
|  | *B. senghasii* G.A.Fischer & A.Sieder | | MAD | FS3970 (SZU) | KJ558727 | KJ558640 | KJ558794 | KJ558974 | KJ558917 | KJ558674 | KJ558869 | KJ558544 |
|  | *B. senghasii* | |  | FS3969 (SZU) | KJ558726 | KJ558638 | KJ558793 | KJ558976 | KJ558916 | KJ558673 | KJ558870 | KJ558546 |
|  | *B.* sp. nov. *‘C’* | | MAD | FS5246 (SZU) | KJ558731 | KJ558604 | KJ558798 | KJ559011 | KJ558921 | KJ558678 | KJ558865 | KJ558581 |
|  | *B.* sp. nov. *‘C’* | |  | FS5385 (SZU) | KJ558733 | KJ558617 | KJ558800 | KJ558998 | KJ558923 | KJ558680 | KJ558863 | KJ558568 |
|  | *B.* sp.nov. *‘D’* | | MAD | FS3967 (SZU) | KJ558725 | KJ558639 | KJ558792 | KJ558975 | KJ558915 | KJ558672 | KJ558871 | KJ558545 |
|  | *B.* sp. nov. *‘E1’* | | MAD | FS6023 (SZU) | KJ558755 | KJ558609 | KJ558823 | KJ559006 | KJ558946 | KJ558703 | KJ558840 | KJ558576 |
|  | *B.* sp. nov. *‘E1’* | |  | FS6027 (SZU) | KJ558756 | - | KJ558824 | KJ558997 | KJ558947 | KJ558704 | KJ558839 | KJ558567 |
|  | *B.* sp. nov. *‘E2’* | | MAD | FS5797 (SZU) | KJ558749 | KJ558606 | KJ558816 | KJ559009 | KJ558939 | KJ558696 | KJ558847 | KJ558579 |
|  | *B. trifarium* Rolfe | | MAD | FS1224 (SZU) | EF196072 | KJ558650 | KJ558782 | KJ558961 | EF200399 | EF202213 | KJ558880 | KJ558532 |
|  | *B. trifarium* | |  | FS1153 (SZU) | KJ558719 | - | KJ558778 | KJ558957 | KJ558908 | KJ558665 | KJ558884 | KJ558528 |
|  | *B. incurvum* Thouars | | **REU**, MAU | REU2 (SZU) | KJ558761 | KJ558594 | KJ558830 | KJ559021 | KJ558952 | KJ558709 | KJ558833 | KJ558591 |
|  | *B. incurvum* | |  | FS1081 (SZU) | KJ558718 | KJ558623 | KJ558776 | KJ558991 | KJ558907 | KJ558664 | KJ558886 | KJ558561 |
|  | *B. malawiense* B.Morris | | AFR | OR146_05 (SZU) | KJ558759 | KJ558630 | KJ558828 | KJ558984 | KJ558950 | KJ558707 | KJ558835 | KJ558554 |
| *Humblotiorchis* Schltr. | | |  |  |  |  |  |  |  |  |  |  |
|  | *B. humblotii* Rolfe | | MAD, REU, SEY, **AFR** | AFRICA8 (WU) | KJ558711 | KJ558655 | KJ558763 | KJ558954 | KJ558900 | KJ558656 | KJ558899 | KJ558525 |
|  | *B. humblotii* | | **MAD,** REU, SEY, AFR | FS1008 (SZU) | EF195986 | KJ558641 | KJ558772 | KJ558973 | EF200402 | EF202216 | KJ558890 | KJ558543 |
| *Alcistachys* Schltr. | | |  |  |  |  |  |  |  |  |  |  |
|  | | *B. variegatum* Thouars | MAD, **REU**, MAU, COM | REU10 (SZU) | KJ558762 | KJ558593 | KJ558831 | KJ559022 | KJ558953 | KJ558710 | KJ558832 | KJ558592 |
|  | | *B. variegatum* | **MAD**, REU,  MAU, COM | FS799 (SZU) | KJ558713 | KJ558627 | KJ558765 | KJ558987 | KJ558902 | KJ558658 | KJ558897 | KJ558557 |
| *Kainochilus* Schltr. | | |  |  |  |  |  |  |  |  |  |  |
|  | | *B. horizontale* Bosser | MAD | AJL148 (SZU) | KJ558712 | - | KJ558764 | KJ558955 | KJ558901 | KJ558657 | KJ558898 | KJ558526 |
| *Inversiflorum* G.A.Fischer & P.J.Cribb in prep. | | | |  |  |  |  |  |  |  |  |  |
|  | | *B. cardiobulbum* Bosser | MAD | FS1641 (SZU) | KJ558721 | KJ558648 | KJ558787 | KJ558966 | KJ558911 | KJ558668 | KJ558876 | KJ558536 |

^*^ Collection area in bold if species is distributed on multiple islands. AFR, Africa; COM, Comores; MAD, Madagascar; MAU, Mauritius; REU, La Réunion; SEY, Seychelles.

^†^ Acronyms of herbaria in which voucher specimens are deposited are in parentheses.
