# Supplementary material for: Multiple independent origins of auto-pollination in tropical orchids (Bulbophyllum) in light of the hypothesis of selfing as an evolutionary dead end
Source: BMC Evol Biol. 2015 Sep 16;15:192. doi: 10.1186/s12862-015-0471-5 (PMC4574068; doi:10.1186/s12862-015-0471-5)
Supplement: Additional file 2: — Newly designed primers for PEPC and pistillata / globosa ( PI ) based on the studies of [63] and [64]. (DOCX 13 kb) [file 12862_2015_471_MOESM2_ESM.docx]

**Additional file 2**

**Gamisch et al. “Multiple independent de novo origins of auto-pollination in tropical orchids (*Bulbophyllum*) conflict with the hypothesis of selfing as an evolutionary dead end”**

**Additional file 6: Newly designed primers for *PEPC* and *pistillata*/*globosa* (*PI*) based on the studies of [63] and [64].**

| Marker | Primer | Sequence 5´ – 3´ |  |
| --- | --- | --- | --- |
| *PEPC* | BULB_PEPC_for4 | AGA GCA GCT GAG AGC CAA CTA | |
|  | BULB_PEPC_rev1.2 | AAA CAT TCA GAG TTG TTA TGT AAG | |
| *Pistillata/globosa* | BULB_Pi1f | GAG ATC AAA CGG ATC GAG AA | |
|  | BULB_Pi1r | TCT RGA TTC GAT CGA TCT CC | |
