# Supplementary figures and images for: Multiple independent origins of auto-pollination in tropical orchids (Bulbophyllum) in light of the hypothesis of selfing as an evolutionary dead end
Source: BMC Evol Biol. 2015 Sep 16;15:192. doi: 10.1186/s12862-015-0471-5 (PMC4574068; doi:10.1186/s12862-015-0471-5)

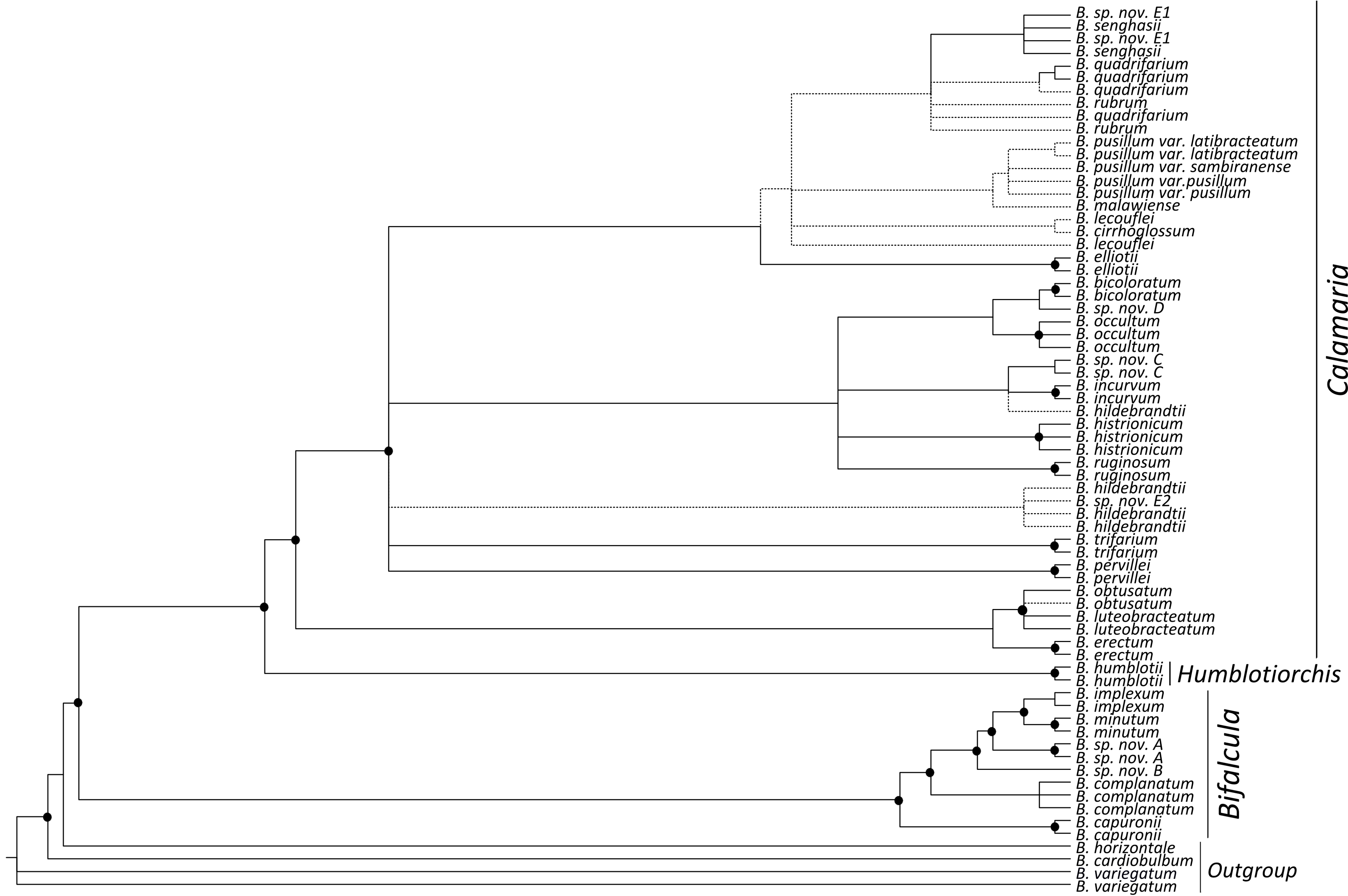

Supplement: Additional file 3: — Majority-rule consensus tree of Madagascan Bulbophyllum clade C from the Bayesian analysis of the plastid five-gene ( atp I –atp H, psb A –trn H, trn D– trn E, trn –trn S, ycf1 ) dataset. Closed circles indicate nodes with Bayesian posterior probability (PP) of 1 and parsimony bootstrap percentage (BP) ≥ 85. Branches only weakly supported by Bayesian analysis (PP ≤ 0.95) are indicated through stippled lines. (TIFF 1199 kb) [file 12862_2015_471_MOESM3_ESM.tif]

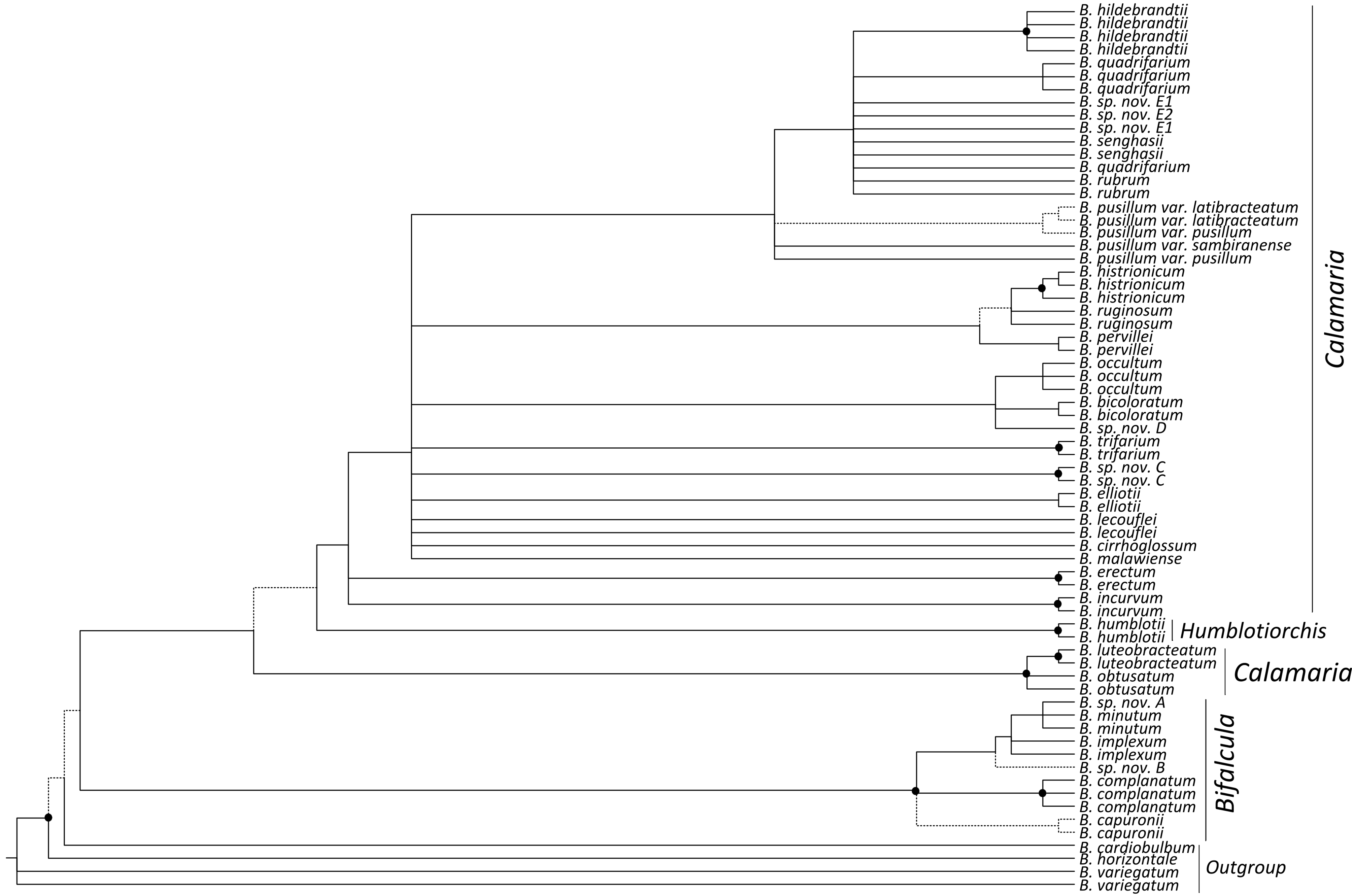

Supplement: Additional file 4: — Majority-rule consensus tree of Madagascan Bulbophyllum clade C from the Bayesian analysis of the nrITS dataset. Closed circles indicate nodes with PP of 1 and BP ≥ 85. Branches only weakly supported by Bayesian analysis (PP ≤ 0.95) are indicated through stippled lines. (TIFF 1102 kb) [file 12862_2015_471_MOESM4_ESM.tif]

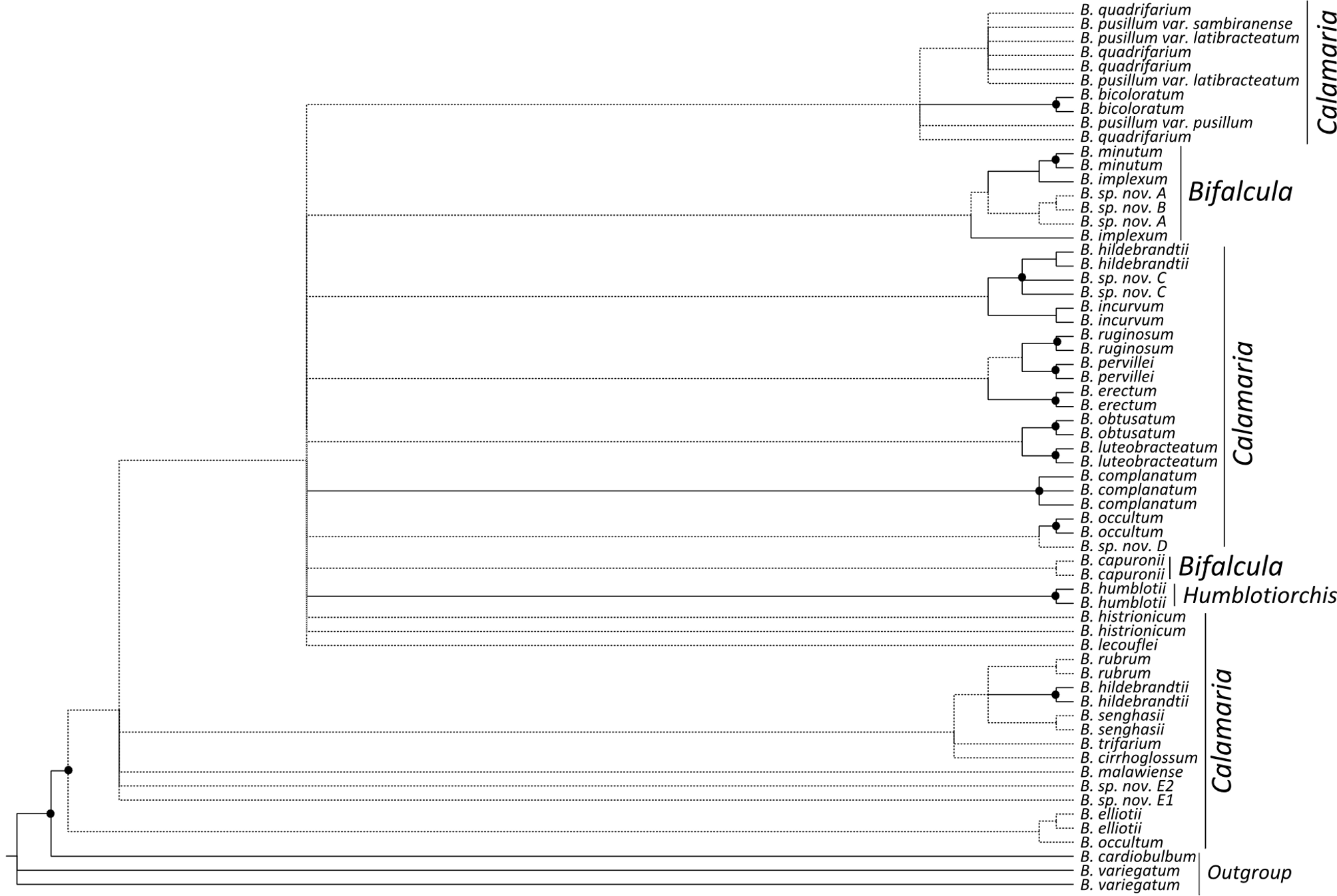

Supplement: Additional file 5: — Majority-rule consensus tree of Madagascan Bulbophyllum clade C from the Bayesian analysis of the single/low copy nuclear marker PEPC. Closed circles indicate nodes with PP of 1 and BP ≥ 85. Branches only weakly supported by Bayesian analysis (PP ≤ 0.95) are indicated through stippled lines. (TIFF 560 kb) [file 12862_2015_471_MOESM5_ESM.tif]

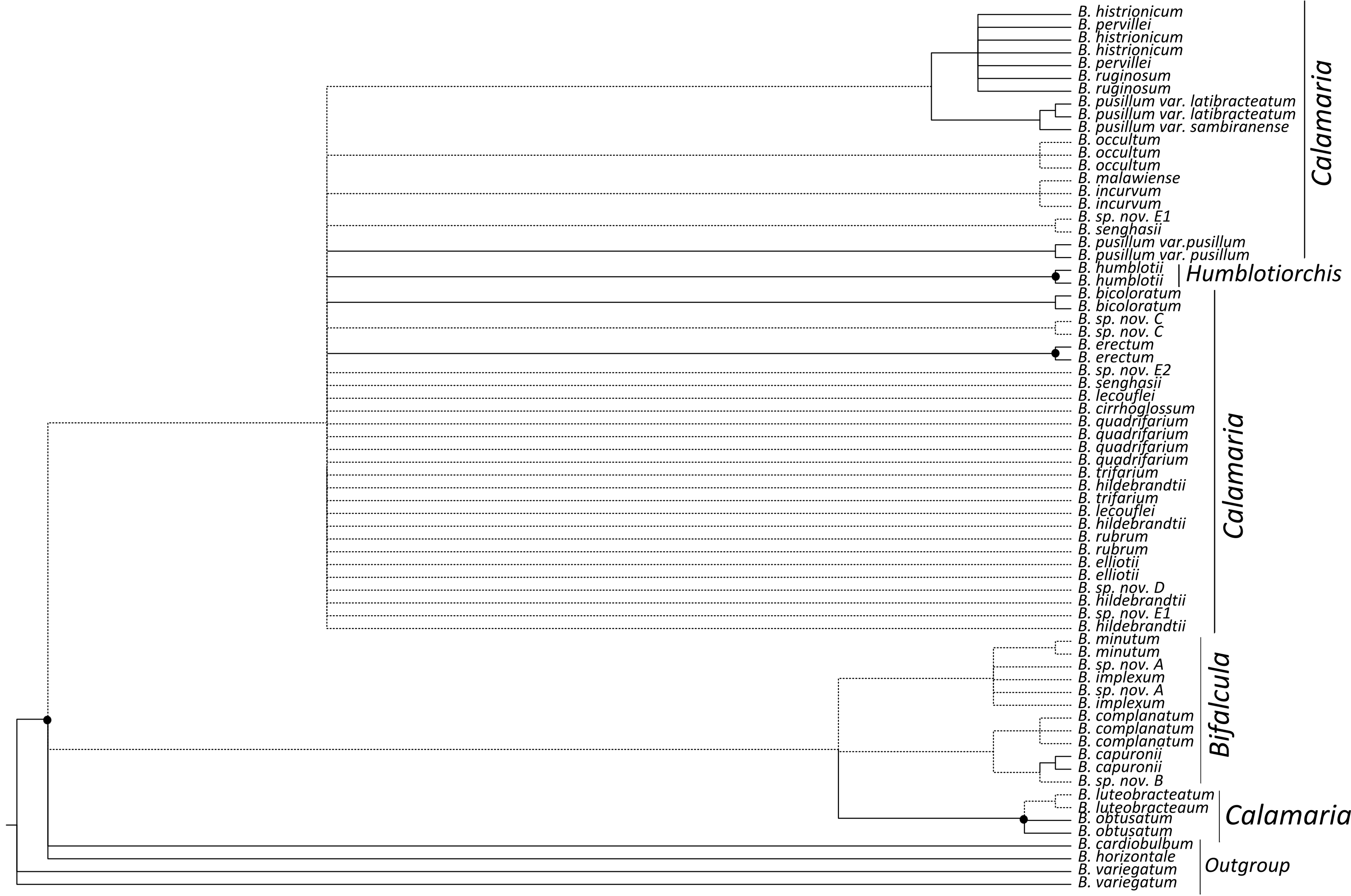

Supplement: Additional file 6: — Majority-rule consensus tree of Madagascan Bulbophyllum clade C from the Bayesian analysis of the single/low copy nuclear marker PI. Closed circles indicate nodes with PP of 1 and BP ≥ 85. Branches only weakly supported by Bayesian analysis (PP ≤ 0.95) are indicated through stippled lines. (TIFF 1626 kb) [file 12862_2015_471_MOESM6_ESM.tif]

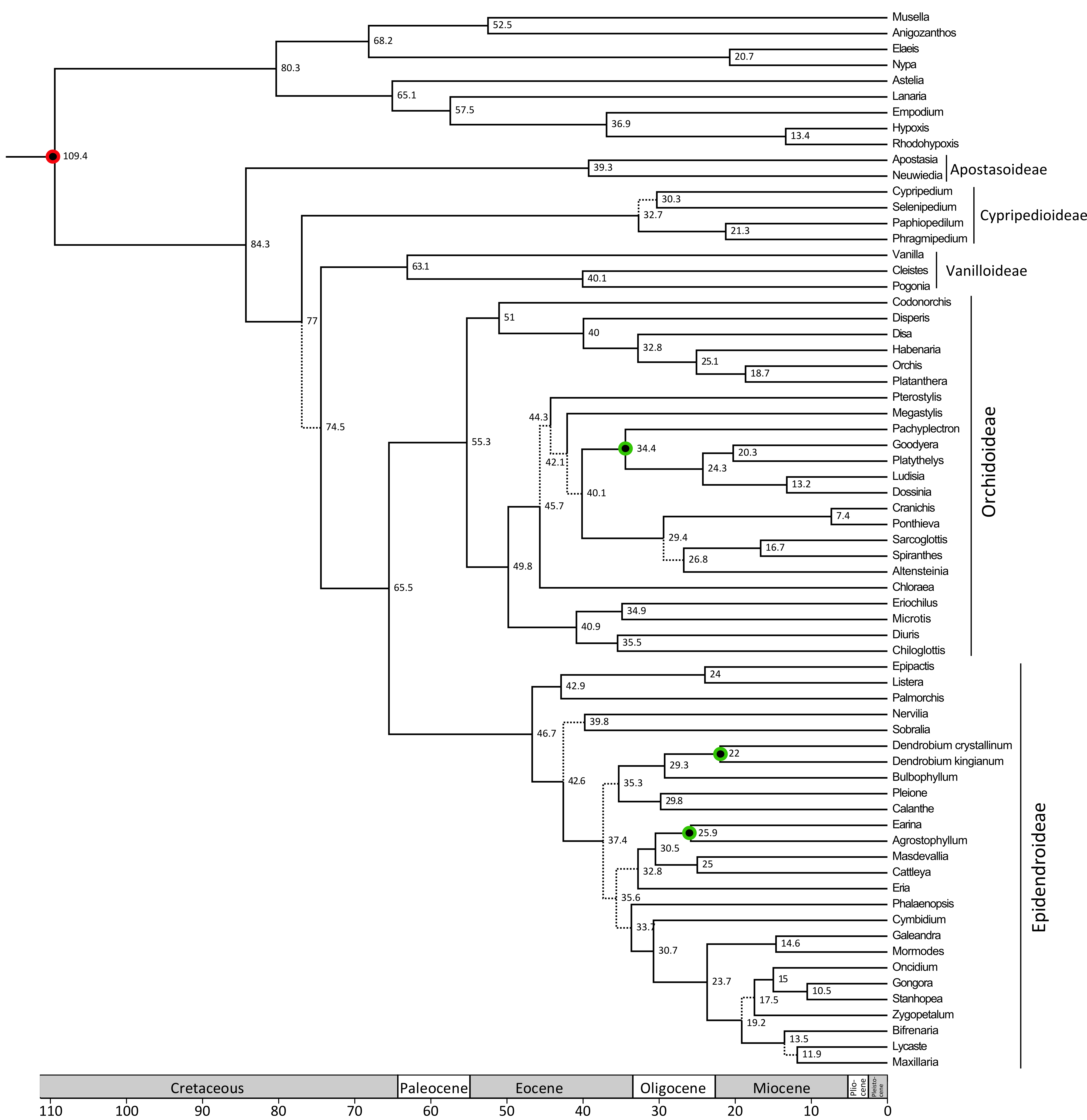

Supplement: Additional file 9: — Time-calibrated phylogeny of the Orchidaceae from the Beast analysis of the plastid two-gene ( mat K, rbc L) dataset, used for the estimation of the stem group age of Bulbophyllum. Numbers at nodes are median ages in million years ago (Ma) (see also [Additional file 12]). Branches only weakly supported by Bayesian analysis (PP < 0.90) are indicated through stippled lines. Colored circles indicate age-constrained nodes (red circle, root node; green circles, fossil-based calibration points). (TIFF 1191 kb) [file 12862_2015_471_MOESM8_ESM.tif]
