# Supplementary material for: Multiple independent origins of auto-pollination in tropical orchids (Bulbophyllum) in light of the hypothesis of selfing as an evolutionary dead end
Source: BMC Evol Biol. 2015 Sep 16;15:192. doi: 10.1186/s12862-015-0471-5 (PMC4574068; doi:10.1186/s12862-015-0471-5)
Supplement: Additional file 7: — Estimating times of divergence: Secondary calibration approach. (DOCX 25 kb) [file 12862_2015_471_MOESM7_ESM.docx]

**Additional file 7**

**Gamisch et al. “Multiple independent de novo origins of auto-pollination in tropical orchids (*Bulbophyllum*) in light of the hypothesis of selfing as an evolutionary dead end”**

**Estimating times of divergence: Secondary calibration approach**

Since there are no fossil records of *Bulbophyllum*, we calibrated our (plastid/nuclear) chronogram of clade C (Figure 3C) using a multi-step secondary calibration approach, which allowed us to include information from fossil-based age estimates derived from recently published family-level plastid DNA (cpDNA) phylogenies in Orchidaceae [132,133]. In a first step, we estimated the stem node age of the genus *Bulbophyllum* based on published cpDNA (*mat*K and *rbc*L) sequences of the Orchidaceae [132,133], representing *Bulbophyllum* (*B. lobbii*) and 56 other orchid genera belonging to all five subfamilies of Orchidaceae*,* plus nine basal genera of Asparagales used as outgroups (one accession per genus, excepting two for *Dendrobium,* 67 accessions in total; see [Additional file 10] for accession numbers). Bayesian searches for tree topologies and node ages of this cpDNA dataset were conducted in Beast version 1.6.1 [36] using GTR+G (*mat*K) and GTR+G+I (*rbc*L) substitution models, selected by jModeltest version 0.1.1 [69], and performing 10 independent Markov chain Monte Carlo (MCMC) runs of 10 million generations each, sampling every 1000 generations. Adopting the fossil-based calibration of [133], we set the age constraints and tree priors to three internal nodes as follows: (i) the crown node age of subtribe Goodyerinae (corresponding to the age of fossil pollinia, 15–20 Ma old, belonging to an extinct species of this taxon; [132]): uniform prior distribution with lower bound of 15 Ma and upper bound of 120 Ma; and two additional calibration points for (ii) the crown node age of *Dendrobium* and (iii) the stem node age of *Earina* (both corresponding to the ages of respective macrofossils, 20–23 Ma old; [134]): uniform prior distribution on the interval 20–120 Ma. The root node age of the tree (corresponding to the oldest known fossil record for Asparagales; see [132]) was set to a uniform prior distribution on the interval 93–120 Ma ([133]; see [Additional files 9,12]). Note that the upper (maximum) age constraint of 120 Ma for the above calibrations corresponds to the oldest known monocot fossils [135]).

In a second step, we determined the crown node age of *Bulbophyllum* clade C. For this purpose, we applied a similar Beast analysis to a comprehensive dataset of nuclear ribosomal (ITS) sequences from 266 species of *Bulbophyllum*, plus those of 11 species of *Dendrobium* used as outgroups ([50,136-138]; Hochschartner *et al.,* unpubl. data; Fischer *et al.,* unpubl. data; see [Additional file 11] for accession numbers). This sampling comprises about 10–15% of the total species diversity of *Bulbophyllum* (ca. 2400 spp.), and includes representatives from across the genus’ pan-tropical range in the Neotropics (with ca. 70% of all known species sampled), Madagascar (ca. 60%), Africa (ca. 55%), Australia (ca. 20%), and Asia (ca. 1.5%) (for estimates of species richness per region see [42,50,138,]. Indels were coded separately using the simple coding method of [139] implemented in Seqstate version 1.4.1 [140]. Accordingly, the dataset was partitioned by nucleotides and indels, with each partition unlinked and set to, respectively, the HKY+G+I model (selected by jModeltest) and a stochastic Dollo model ([141,142]; A. Rambaut A. and M. Suchard, pers. comm.). Three independent MCMC runs were performed with 50 million generations each, sampling every 5000 generations. Altogether four tree priors were set for this ITS phylogeny (see [Additional file 8,13]): (i) the age for the root node of the tree (corresponding to the median stem node age of the *Bulbophyllum/Dendrobium* clade approximated from the cpDNA phylogeny of Orchidaceae see [Additional file 9,12]): normal prior distribution with mean 30.17 Ma and standard deviation of 3.48, giving a 95% confidence interval (CI) of 23.3–36.99 Ma; (ii) the crown node age of *Dendrobium*: uniform prior distribution with a lower bound of 20 Ma ([133]; see above) and an upper bound of 36.99 Ma (based on the CI above); (iii) the divergence time of the inferred sister species (Figures 2–3) *Bulbophyllum* sp. nov*.* ‘*C’* (Madagascar) and *B. incurvum* (endemic to La Réunion/Mauritius): uniform prior distribution with an upper bound of 7.8 Ma (corresponding to the age of Mauritius; [140]); and (iv) the divergence time of *B. molossus* (Madagascar) and *B. macrocarpum* (endemic to La Réunion, [49]): uniform prior distribution with an upper (maximum) bound of 2.2 Ma (corresponding to the age of La Réunion; [144]). Although the latter two constraints may be questionable (because island ages provide only maximum ages of divergence), the upper prior distributions specified should sufficiently account for the likelihood that the ancestors of the island endemics arrived at an unspecified time after oceanic island formation [145] for the successful application of island ages used as calibration points in a similar geographical context). In a third and final Beast analysis, we point-calibrated the age of the root node of our combined plastid/nuclear chronogram of *Bulbophyllum* clade C (Figure 3C) as a narrow normal prior distribution with mean 5.32 Ma and standard deviation of 10^-4^, which corresponds to the median crown node age of clade C approximated from the ITS phylogeny of *Bulbophyllum* (see [Additional file 8,13]).

For each beast analysis described above, a birth-death process was specified as tree prior and an uncorrelated lognormal relaxed clock was assumed [80]. After removal of the first 15% of the MCMC samples as burn-in, results of the independent runs were combined using LogCombiner version 1.6.1 [36] and inspected in Tracer version 1.5 [71] to confirm convergence of the chain to stationary and assess sampling adequacy. The combined effective sampling sizes (ESSs) for all parameters were >200 indicating a sufficient level of sampling. Resulting chronograms were visualized in figtree version 1.3.1 (http: //tree.bio.ed.ac.uk/software/figtree/). As indicated above, we used uniform bounded priors for all fossil-based and biogeographic (island age) constraints and normal distributed priors for all secondary (indirect) calibration points. The usage of uniform distributed priors with minimum bounds are regarded as a very conservative way to incorporate fossil information in molecular phylogenetic studies, while normal distributed priors are well suited to reflect the uncertainty of date estimates derived from independent molecular dating studies [146].

**Additional References**

132. Ramírez SR, Gravendeel B, Singer RB, Marshall CR, Pierce NE: **Dating the origin of the Orchidaceae from a fossil orchid with its pollinator.** *Nature* 2007, **448**:1042-1045.

133. Gustafsson ALS, Verola CF, Antonelli A: **Reassessing the temporal evolution of orchids with new fossils and a Bayesian relaxed clock with implications for the diversification of the rare South American genus *Hoffmannseggella* (Orchidaceae: Epidendroideae).** *BMC Evol Biol* 2010, **10**:177.

134. Conran JG, Bannister JM, Lee ED: **Earliest orchid macrofossils: early Miocene *Dendrobium* and *Earina* (Orchidaceae: Epidendroideae) from New Zealand.** *Am J Bot* 2009, **96**:466-474.

135. Friis EM, Pedersen KR, Crane PR: **Araceae from the Early Cretaceous of Portugal: evidence on the emergence of monocotyledons.** *Proc Natl Acad Sci USA* 2004, **101**:16565-16570.

136. Tsai CC, Peng CI, Huang SC, Huang PL, Chou CH: **Determination of the genetic relationship of *Dendrobium* species (Orchidaceae) in Taiwan based on the sequence of the internal transcribed spacer of ribosomal DNA.** *Sci Hort* 2004, **101**:315-325.

137. Yuan ZQ, Zhang JY, Liu T: **Phylogenetic relationship of China *Dendrobium* species based on the sequence of the internal transcribed spacer of ribosomal DNA.** *Biol Plantarum* 2009, **53**:155-158.

138. Smidt EC, Borba EL, Gravendeel B, Fischer GA, Berg CVD: **Molecular phylogeny of the Neotropical sections of *Bulbophyllum* (Orchidaceae) using nuclear and plastid spacers.** *Taxon* 2011, **60**:1050-1064.

139. Simmons MP, Ochoterena H: **Gaps as characters in sequence-based phylogenetic analyses.** *Syst Biol* 2000, **49**:369-381.

140. Müller K: **SeqState - primer design and sequence statistics for phylogenetic DNA data sets.** *Appl Bioinformatics* 2005, **4**:65-69.

141. Alekseyenko AV, Lee CJ, Suchard MA: **Wagner and Dollo: a stochastic duet by composing two parsimonious solos.** *Syst Biol* 2008, **57**:772-784.

142. Pyron RA: **Divergence time estimation using fossils as terminal taxa and the origins of Lissamphibia.** *Syst Biol* 2011, **60**:466-481.

143. McDougall I, Chamalaun FH: **Isotopic dating and geomagnetic polarity studies on volcanic rocks from Mauritius Indian Ocean.** *Geol Soc Am Bull* 1969, **80**:1419-1442.

144. Quidelleur X, Holt JW, Salvany T, Bouquerel H: **New K-Ar ages from LaMontagne massif Réunion Island (Indian Ocean) supporting two geomagnetic events in the time period 2.2-2.0 Ma.** *Geophys J Int* 2010, **182**:699-710.

145. Jønsson KA, Bowie RCK, Nylander JAA, Christidis L, Norman JA, Fjeldsa J: **Biogeographical history of cuckoo-shrikes (Aves: Passeriformes): transoceanic colonization of Africa from Australo-Papua.** *J Biogeogr* 2010, **37**:1767-1781.

146. Ho SY-W: **Calibrating molecular estimates of substitution rates and divergence times in birds.** *J Avian Biol* 2007, **38**:409-414.
