# Supplementary material for: Multiple independent origins of auto-pollination in tropical orchids (Bulbophyllum) in light of the hypothesis of selfing as an evolutionary dead end
Source: BMC Evol Biol. 2015 Sep 16;15:192. doi: 10.1186/s12862-015-0471-5 (PMC4574068; doi:10.1186/s12862-015-0471-5)
Supplement: Additional file 10: — GenBank (NCBI) accession numbers of plastid DNA ( mat K, rbc L) sequences of Orchidaceae used to estimate the stem node age of the genus Bulbophyllum. (DOCX 20 kb) [file 12862_2015_471_MOESM10_ESM.docx]

**Additional file 10**

**Gamisch et al. “Multiple independent de novo origins of auto-pollination in tropical orchids (*Bulbophyllum*) in light of the hypothesis of selfing as an evolutionary dead end”**

**Additional file 10: GenBank (NCBI) accession numbers of plastid DNA (*mat*K, *rbc*L) sequences of Orchidaceae used to estimate the stem node age of the genus *Bulbophyllum*.**

| Subfamily/genus (species) | | GenBank accession number | |
| --- | --- | --- | --- |
|  |  | *mat*K | *rbc*L |
| Apostasioideae | |  |  |
|  | *Apostasia* | AY557214.1 | Z73705 |
|  | *Neuwiedia* | AY557211.1 | AF074200 |
| Vanilloideae | |  |  |
|  | *Cleistes* | AJ310006 | AF074128 |
|  | *Pogonia* | AJ310055 | AF074221 |
|  | *Vanilla* | AF263687 | AF074242 |
| Cypripedioideae | |  |  |
|  | *Cypripedium* | AF263649 | AF074142 |
|  | *Paphiopedilum* | AY368379 | AF074208 |
|  | *Phragmipedium* | AY368380 | AF074213 |
|  | *Selenipedium* | AY368381.1 | AF074227 |
| Orchidoideae | |  |  |
|  | *Altensteinia* | AJ309989 | AF074105 |
|  | *Chiloglottis* | AJ310003 | AF074124 |
|  | *Chloraea* | AJ310005 | AF074125 |
|  | *Codonorchis* | AJ310007 | AY368338 |
|  | *Cranichis* | AJ310013 | AF074137 |
|  | *Disa* | AF263654 | AF274006 |
|  | *Disperis* | AY370652.1 | AY370651 |
|  | *Diuris* | AF263655 | AF074152 |
|  | *Dossinia* | AJ543947.1 | AJ542405 |
|  | *Eriochilus* | AJ310028 | AF074166 |
|  | *Goodyera* | AF263663 | AF074174 |
|  | *Habenaria* | AJ310036 | AF074177 |
|  | *Ludisia* | AJ543911.1 | AJ542395 |
|  | *Megastylis* | AJ310042 | AF074191 |
|  | *Microtis* | AJ310045 | AF074194 |
|  | *Orchis* | AY368385 | AF074203 |
|  | *Pachyplectron* | AJ310051.1 | AF074205 |
|  | *Platanthera* | AF263678 | AF074215 |
|  | *Platythelys* | AY368386.1 | AF074216 |
|  | *Ponthieva* | AJ310056 | AF074223 |
|  | *Pterostylis* | AJ310062 | AF074224 |
|  | *Sarcoglottis* | AJ310068 | AY368347 |
|  | *Spiranthes* | AF263682 | AF074229 |
| Lower Epidendroideae | |  |  |
|  | *Epipactis* | AF263659 | Z73707 |
|  | *Listera* | AF263668 | AF074184 |
|  | *Nervilia* | AY368420 | AF074199 |
|  | *Palmorchis* | AJ310052 | AF074206 |
|  | *Sobralia* | AF263681 | AF074228 |
| Higher Epidendroideae | |  |  |
|  | *Agrostophyllum* | AY368391.1 | AF518054.1 |
|  | *Bifrenaria* | AY368394 | AF074112 |
|  | *Bulbophyllum* | AY368395.1 | AF074115.1 |
|  | *Calanthe* | AF263632 | AF264159 |
|  | *Cattleya* | AF263638 | AF074122 |
|  | *Cymbidium* | AF263648 | AF074141 |
|  | *Dendrobium* (*D. crystallinum*) | AF445447.1 | D58407.1 |
|  | *Dendrobium* (*D. kingianum*) | AF263651.1 | AF074146.1 |
|  | *Earina* | AF263656.1 | AF074155.1 |
|  | *Eria* | AF263660 | AF074164 |
|  | *Galeandra* | AY368408 | AF074171 |
|  | *Gongora* | AY368409 | AY368358 |
|  | *Lycaste* | AF263669 | AF074185 |
|  | *Masdevallia* | AY368416 | AF074189 |
|  | *Maxillaria* | AF239427 | AF074190 |
|  | *Mormodes* | AY368417 | AF074196 |
|  | *Oncidium* | AY368423 | AF074201 |
|  | *Phalaenopsis* | AF263677 | AF074211 |
|  | *Pleione* | AF263679 | AF264173 |
|  | *Stanhopea* | AY368430 | AF074230 |
|  | *Zygopetalum* | AF263689 | AF074246 |
| Outgroup | |  |  |
|  | *Astelia* | AY368372.1 | Z77261 |
|  | *Anigozanthos* | AB088796.1 | AJ404843.1 |
|  | *Elaeis* | EU016887.1 | AY012509.1 |
|  | *Empodium* | AY368374.1 | Y14987.1 |
|  | *Hypoxis* | AY368375.1 | Z73702 |
|  | *Lanaria* | AY368376.1 | Z77313 |
|  | *Musella* | AF478909.1 | AF243844.1 |
|  | *Nypa* | AF543743.1 | M81813.1 |
|  | *Rhodohypoxis* | AY368377.1 | Z77280 |
