# Supplementary material for: Multiple independent origins of auto-pollination in tropical orchids (Bulbophyllum) in light of the hypothesis of selfing as an evolutionary dead end
Source: BMC Evol Biol. 2015 Sep 16;15:192. doi: 10.1186/s12862-015-0471-5 (PMC4574068; doi:10.1186/s12862-015-0471-5)
Supplement: Additional file 11: — List of 277 accessions of nrDNA (ITS) sequences of Bulbophyllum and Dendrobium used to estimate the crown node age of Madagascan Bulbophyllum clade C. (DOCX 74 kb) [file 12862_2015_471_MOESM11_ESM.docx]

**Additional file 11**

**Gamisch et al. “Multiple independent de novo origins of auto-pollination in tropical orchids (*Bulbophyllum*) in light of the hypothesis of selfing as an evolutionary dead end”**

**Additional file 11: List of 277 accessions of nrDNA (ITS) sequences of *Bulbophyllum* and *Dendrobium* used to estimate the crown node age of Madagascan *Bulbophyllum* clade C.** Note, this list includes 190 GenBank (NCBI) accessions plus 87 yet unpublished sequences (Fischer et al. unpubl. data; Hochschartner et al. unpubl. data) for which voucher information is provided.

| Genus/species | | GenBank accession number (nrITS) | Voucher number ^*^ | Origin | Source |
| --- | --- | --- | --- | --- | --- |
| *Bulbophyllum* | |  |  |  |  |
|  | *B. adiamantinum* Brade | GQ339691.1 | - | South America | [135] |
|  | *B. affine* Lindl. | EF195916.1 | - | Asia | [50] |
|  | *B. afzelii* Schltr. | - | FS2526 (SZU) | Madagascar | Fischer et al. unpubl. data |
|  | *B. aggregatum* Bosser | EF195951.1 | - | Madagascar | [50] |
|  | *B. alexandrae* Schltr. | EF195952.1 | - | Madagascar | [50] |
|  | *B. alinae* D.Szlachetko & T.Olszewski | - | DSS1474 (SZU) | Africa | Fischer et al. unpubl. data |
|  | *B. alleizettei* Schltr. | EF195953.1 | - | Madagascar | [50] |
|  | *B. alsiosum* Ames | EF195917.1 | - | Asia | [50] |
|  | *B. ambatoavense* Bosser | EF195954.1 | - | Madagascar | [50] |
|  | *B. amoenum* Bosser | EF195955.1 | - | Madagascar | [50] |
|  |  |  |  |  |  |
|  | *B. amphorimorphum* H.Perrier | EF196069.1 | - | Madagascar | [50] |
|  | *B. analamazoatrae* Schltr. var. nov. | EF195957.1 | - | Madagascar | [50] |
|  | *B. anjozorobeense* Bosser | EF195958.1 | - | Madagascar | [50] |
|  | *B. ankaizinense* (Jum. & H.Perrier) Schltr. | EF195959.1 | - | Madagascar | [50] |
|  | *B. approximatum* Ridl. | EF195960.1 | - | Madagascar | [50] |
|  | *B. atropurpureum* Barb.Rodr. | GQ339706.1 | - | South America | [135] |
|  | *B. aubrevillei* Bosser | EF195962.1 | - | Madagascar | [50] |
|  | *B. auriculatum* Garay | - | OR145_05 (SZU) | Asia | Hochschartner et al. unpubl. data |
|  | *B. auriflorum* H.Perrier | EF195963.1 | - | Madagascar | [50] |
|  | *B. barbigerum* Lindl. | EF195918.1 | - | Africa | [50] |
|  | *B. aff. baronii* Ridl. | EF195948.1 | - | Madagascar | [50] |
|  | *B. bathieanum* Schltr. | - | FS5219 (SZU) | Madagascar | Fischer et al. unpubl. data |
|  | *B. bicoloratum* Schltr. | EF195964.1 | - | Madagascar | [50] |
|  | *B. bidentatum* (Barb.Rodr. ) Cogn. | GQ339701.1 | - | South America | [135] |
|  | *B. bifarium* Hook.f. | - | VD&S702 (SZU) | Africa | Fischer et al. unpubl. data |
|  | *B. biflorum* Teijsm. & Binn. | EF195919.1 | - | Asia | [50] |
|  | *B. boudetianum* Fraga | GQ339723.1 | - | South America | [135] |
|  | *B. bowkettae* F.M.Bailey | - | OR6 (SZU) | Australia | Fischer et al. unpubl. data |
|  | *B. bracteolatum* Lindl. | EF195920.1 | - | South America | [135] |
|  | *B. bryophytoides* G.A.Fischer & J.Andriantiana | - | FS2595 (SZU) | Madagascar | Fischer et al. unpubl. data |
|  | *B. burfordiense* Garay, Hamer & Siegerist | AY273716.1 | - | Asia | [133] |
|  | *B. callosum* Bosser | - | OR183_03 (SZU) | Madagascar | Fischer et al. unpubl. data |
|  | *B. calvum* Summerh. | - | VD&S672 (SZU) | Africa | Fischer et al. unpubl. data |
|  | *B. calyptratum*  Kraenzl. | - | OR155_05 (SZU) | Africa | Fischer et al. unpubl. data |
|  | *B. calyptropus* Schltr. | EF195965.1 | - | Madagascar | [50] |
|  | *B. cameronense* Garay, Hamer & Siegerist | - | OR90_99 (SZU) | Asia | Hochschartner et al. unpubl. data |
|  | *B. campos-portoi* Brade | GQ339721.1 | - | South America | [135] |
|  | *B. cantagallense* Barb.Rodr. | GQ339722.1 | - | South America | [135] |
|  | *B. capituliflorum* Rolfe | - | DSS1471 (SZU) | Africa | Fischer et al. unpubl. data |
|  | *B. capuronii* Bosser | EF195966.1 | - | Madagascar | [50] |
|  | *B. carassense* R.C.Mota | GQ339717.1 | - | South America | [135] |
|  | *B. cardiobulbum* Bosser | EF195967.1 | - | Madagascar | [50] |
|  | *B. chloroglossum* Rchb.f. &  Warm. | GQ339694.1 | - | South America | [135] |
|  | *B. aff. chloropterum* Rchb.f. | EF195921.1 | - | South America | [135] |
|  | *B. ciliatilabrum* H.Perrier | EF195968.1 | - | Madagascar | [50] |
|  | *B. ciluliae* Bianch. & J.A.N.Bat. | GQ339698.1 | - | South America | [135] |
|  | *B. cirrhoglossum* H.Perrier | KJ558728 | - | Madagascar | present study |
|  | *B. cirrhosum* L.O.Williams | GQ339685.1 | - | South America | [135] |
|  | *B. clandestinum* Lindl. | JF706719.1 | - | Asia | [135] |
|  | *B. claptonense* Rolfe | - | OR160_05 (SZU) | Asia | Hochschartner et al. unpubl. data |
|  | *B. coccinatum* H.Perrier | - | FS4224 (SZU) | Madagascar | Fischer et al. unpubl. data |
|  | *B. cochleatum* Lindl. | - | OR50 (SZU) | Africa | Fischer et al. unpubl. data |
|  | *B. cochleatum* var. *tenuicaule* (Lindl.) J.J.Verm. | - | Bthomense (SZU) | Africa | Fischer et al. unpubl. data |
|  | *B. cocoinum* Bateman ex Lindl. | - | OR714_03 (SZU) | Africa | Fischer et al. unpubl. data |
|  | *B. colubrinum* (Rchb.f.) Rchb.f. | - | DSS851 (SZU) | Africa | Fischer et al. unpubl. data |
|  | *B. comatum* var. *inflatum* (Rolfe) J.J.Verm. | - | 1799 (SZU) | Africa | Fischer et al. unpubl. data |
|  | *B. complanatum* H.Perrier | EF633602.1 | - | Madagascar | [50] |
|  | *B. conchidioides* Ridl. | EF195969.1 | - | Madagascar | [50] |
|  | *B. coriophorum* Ridl. | EF195970.1 | - | Madagascar | [50] |
|  | *B.* *coweniorum* J.J.Verm. & P.O'Byrne | - | OR222_98 (SZU) | Asia | Hochschartner et al. unpubl. data |
|  | *B. cribbianum* Toscano | EF195922.1 | - | South America | [50] |
|  | *B. cumingii* (Lindl.) Rchb.f. | EF195923.1 | - | Asia | [50] |
|  | *B. cyclanthum* Schltr. | EF195971.1 | - | Madagascar | [50] |
|  | *B. dearei* (Hort.) Rchb.f. | EF195924.1 | - | Asia | [50] |
|  | *B. debile* Bosser | - | FS4070 (SZU) | Madagascar | Fischer et al. unpubl. data |
|  | *B. densum* Thouars | - | REU4 (SZU) | Madagascar, Réunion | Fischer et al. unpubl. data |
|  | *B. devium* J.B.Comber | - | OR1521 (SZU) | Africa | Fischer et al. unpubl. data |
|  | *B. divaricatum* H.Perrier | - | OR1319 (SZU) | Madagascar | Fischer et al. unpubl. data |
|  | *B. edentatum* H.Perrier | EF195972.1 | - | Madagascar | [50] |
|  | *B. elliotii* Rolfe | EF195976.1 | - | Madagascar | [50] |
|  | *B. emiliorum* Ames & Quisumb. | EF195926.1 | - | Asia | [50] |
|  | *B. encephalodes* Summerh. | - | OR251_09 (SZU) | Africa | Fischer et al. unpubl. data |
|  | *B. epiphytum* Barb.Rodr. | GQ339693.1 | - | South America | [135] |
|  | *B. erectum* Thouars | KJ558717 | - | Madagascar | present study |
|  | *B. exaltatum* Lindl. | GQ339715.1 | - | South America | [135] |
|  | *B. exiguum* F.Muell. | - | OR8 (SZU) | Australia | Fischer et al. unpubl. data |
|  | *B. facetum Garay,* Hamer & Siegerist | - | OR619_99 (SZU) | Asia | Hochschartner et al. unpubl. data |
|  | *B. falcatum* (Lindl.) Rchb.f | EF195927.1 | - | Africa | [50] |
|  | *B. filifolium* Borba & E.C. Smidt | GQ339699.1 | - | South America | [135] |
|  | *B. florulentum* Schltr. | EF195996.1 | - | Madagascar | [50] |
|  | *B. forsythianum* Kraenzl. | EF196063.1 | - | Madagascar | [50] |
|  | *B. francoisii* H.Perrier | EF195977.1 | - | Madagascar | [50] |
|  | *B. fuscum* Lindl. | - | DSS914 (SZU) | Africa | Fischer et al. unpubl. data |
|  | *B. gladiatum* Lindl. | GQ339718.1 | - | South America | [135] |
|  | *B. glutinosum* (Barb.Rodr.) Cogn. | EF195928.1 | - | South America | [50] |
|  | *B. graciliscapum* H.Perrier | EF196010.1 | - | Madagascar | [50] |
|  | *B. hamatipes* J.J.Sm. | EF195929.1 | - | Asia | [50] |
|  | *B. hamelinii* W.Watson | EF195979.1 | - | Madagascar | [50] |
|  | *B. hapalanthos* Garay | EF195980.1 | - | Madagascar | [50] |
|  | *B. henrici* Schltr. var. *rectangulare* H.Perrier | EF195981.1 | - | Madagascar | [50] |
|  | *B. hiepii* Aver. | - | OR425 (SZU) | Asia | Hochschartner et al. unpubl. data |
|  | *B. hildebrandtii* Rchb.f. | EF195983.1 | - | Madagascar | [50] |
|  | *B. histrioncum* Rchb.f. ex G.A.Fischer & P.J.Cribb | EF196062.1 | - | Madagascar | [50] |
|  | *B. hoehnei* E.C.Smidt & Borba | GQ339700.1 | - | South America | [135] |
|  | *B. horizontale* Bosser | EF195984.1 | - | Madagascar | [50] |
|  | *B. humbertii* Schltr. | EF195985.1 | - | Madagascar | [50] |
|  | *B. humblottii* Rolfe | EF195986.1 | - | Madagascar | [50] |
|  | *B.* hyalosemoides J.J. Verm & O'Byrne | - | SBGO4903 (SZU) | Asia | Hochschartner et al. unpubl. data |
|  | *B. imbricatum* Lindl. | - | DSS906 (SZU) | Africa | Fischer et al. unpubl. data |
|  | *B. imerinense* Schltr. | EF195987.1 | - | Madagascar | [50] |
|  | *B. imerinense* Schltr. | - | FS2991 (SZU) | Madagascar | Fischer et al. unpubl. data |
|  | *B. incurvum* Thouars | KJ558718 | - | Réunion | present study |
|  | *B. insectiferum* Barb.Rodr. | GQ339692.1 | - | South America | [135] |
|  | *B. insolitum* Bosser | EF196019.1 | - | Madagascar | [50] |
|  | *B. intertextum* Lindl. | EF195930.1 | - | Madagascar | [50] |
|  | *B. jaapii* Szlatch. & Olszweski | - | VD&S668 (SZU) | Africa | Fischer et al. unpubl. data |
|  | *B. jackyi* G.A.Fischer, A.Sieder  & P.J.Cribb | EF196020.1 | - | Madagascar | [50] |
|  | *B. josephi* (Kuntze) Summerh. | - | DSS1220 (SZU) | Africa | Fischer et al. unpubl. data |
|  | *B. jumelleanum* Schltr. | - | OR1369 (SZU) | Madagascar | Fischer et al. unpubl. data |
|  | *B. kautskyi* Toscano | GQ339705.1 | - | South America | [135] |
|  | *B. kupense* P.J.Cribb & B.J.Pollard | - | VD&S712 (SZU) | Africa | Fischer et al. unpubl. data |
|  | *B. lakatoense* Bosser | - | OR84_05 (SZU) | Madagascar | Fischer et al. unpubl. data |
|  | *B. latipetalum* H.Perrier | EF196067.1 | - | Madagascar | [50] |
|  | *B. leandrianum* H.Perrier | EF196021.1 | - | Madagascar | [50] |
|  | *B. lecouflei* Bosser | EF196029.1 | - | Madagascar | [50] |
|  | *B. leopardinum* (Wall.) Lindl. | - | OR1530 (SZU) | Asia | Fischer et al. unpubl. data |
|  | *B. leptostachyum* Schltr. | EF196005.1 | - | Madagascar | [50] |
|  | *B. liparidioides* Schltr. | EF196022.1 | - | Madagascar | [50] |
|  | *B. lizae* J.J.Verm. | - | 1779 (SZU) | Africa | Fischer et al. unpubl. data |
|  | *B. lobbii* Lindl. | - | 960117 (SZU) | Asia | Hochschartner et al. unpubl. data |
|  | *B. longiflorum* Thouars | EF196023.1 | - | Madagascar | [50] |
|  | *B. longivaginans* H.Perrier | EF196025.1 | - | Madagascar | [50] |
|  | *B. lucidum* Schltr. | EF195988.1 | - | Madagascar | [50] |
|  | *B. lupulinum* Lindl. | EF195932.1 | - | Africa | [50] |
|  | *B. luteobracteatum* Jum. & H.Perrier | - | OR1535 (SZU) | Madagascar | Fischer et al. unpubl. data |
|  | *B. lyperocephalum* Schltr. | - | FS2680 (SZU) | Madagascar | Fischer et al. unpubl. data |
|  | *B. macphersonii* Rupp | - | OR7 (SZU) | Australia | Fischer et al. unpubl. data |
|  | *B. macranthum* Lindl. | EF195933.1 | - | Asia | [50] |
|  | *B. macrocarpum* Frapp. ex Cordem. | - | REU5 (SZU) | Madagascar, Réunion | Fischer et al. unpubl. data |
|  | *B. malachadenia* (Lindl.) Cogn. | GQ339708.1 | - | South America | [135] |
|  | *B. malawiense* Morris | KJ558759 | - | Africa | present study |
|  | *B. manarae* Foldats | GQ339704.1 | - | South America | [135] |
|  | *B. mangenotii* Bosser | - | OR75_05 (SZU) | Madagascar | Fischer et al. unpubl. data |
|  | *B. marovoense* H.Perrier | EF196027.1 | - | Madagascar | [50] |
|  | *B. masoalanum* Schltr. | - | OR1346 (SZU) | Madagascar | Fischer et al. unpubl. data |
|  | *B. maximum* (Lindl.) Rchb.f. | - | DSS848 (SZU) | Africa | Fischer et al. unpubl. data |
|  | *B. mayombeense* Garay | EF195934.1 | - | Africa | [50] |
|  | *B. mediocre* Summerh. | - | 2099LA (SZU) | Africa | Fischer et al. unpubl. data |
|  | *B. melleum* H.Perrier | EF196030.1 | - | Madagascar | [50] |
|  | *B. melloi* Pabst | GQ339719.1 | - | South America | [135] |
|  | *B. membranifolium* Hook.f. | EF195935.1 | - | Asia | [50] |
|  | *B. membranifolium* Hook.f. | - | OR575_98 (SZU) | Asia | Hochschartner et al. unpubl. data |
|  | *B. mentosum* Barb.Rodr. | GQ339690.1 | - | South America | [135] |
|  | *B. meridense* Rchb.f. | GQ339712.1 | - | South America | [135] |
|  | *B. micranthum* Barb.Rodr. | GQ339697.1 | - | South America | [135] |
|  | *B. microglossum* Ridl. | - | OR163_99 (SZU) | Asia | Hochschartner et al. unpubl. data |
|  | *B. micropetaliforme* Leite | GQ339709.1 | - | South America | [135] |
|  | *B. minutissimum* (F. Muell.) F.Muell. | - | Australia2 (SZU) | Australia | Fischer et al. unpubl. data |
|  | *B. minutum* Thouars | EF196031.1 | - | Madagascar | [50] |
|  | *B. minutum* Thouars | EF196061.1 | - | Madagascar | [50] |
|  | *B. molossus* Rchb.f. | EF196032.1 | - | Madagascar | [50] |
|  | *B. monanthum* (Kuntze) J.J.Sm. | - | OR144 (SZU) | Asia | Hochschartner et al. unpubl. data |
|  | *B. moratii* Bosser | - | FS4250 (SZU) | Madagascar | Fischer et al. unpubl. data |
|  | *B. mucronifolium* Rchb.f. & Warm. | GQ339695.1 | - | South America | [135] |
|  | *B. muscicola* Schltr. | EF196033.1 | - | Madagascar | [50] |
|  | *B. nagelii* L.O.Williams | GQ339720.1 | - | South America | [135] |
|  | *B. namoronae* Bosser | - | OR1341 (SZU) | Madagascar | Fischer et al. unpubl. data |
|  | *B. napellii* Lindl. | GQ339711.1 | - | South America | [135] |
|  | *B. newportii* (F.M.Bailey) Rolfe | JF706720.1 | - | Australia | [135] |
|  | *B. nigriflorum* H.Perrier | EF195993.1 | - | Madagascar | [50] |
|  | *B. nigritianum* Rendle | - | DSS1406 (SZU) | Africa | Fischer et al. unpubl. data |
|  | *B. nitens* Jum. & H.Perrier | EF196034.1 | - | Madagascar | [50] |
|  | *B. nutans* (Thouars) Thouars | EF196035.1 | - | Madagascar | [50] |
|  | *B. obtusatum* (Jum. & H.Perrier) Schltr*.* | EF196039.1 | - | Madagascar | [50] |
|  | *B. occlusum* Ridl*.* | EF196040.1 | - | Madagascar | [50] |
|  | *B. occultum* Thouars | EF196041.1 | - | Madagascar | [50] |
|  | *B. ochrochlamys* Schltr. | EF196042.1 | - | Madagascar | [50] |
|  | *B. ophiuchus* Ridl. | EF195990.1 | - | Madagascar | [50] |
|  | *B. orectopetalum* Garay, Hamer & Siegerist | EF195936.1 | - | Asia | [50] |
|  | *B. oreodorum* Schltr. | EF196043.1 | - | Madagascar | [50] |
|  | *B. oreonastes* Rchb.f. | - | DSS968 (SZU) | Africa | Fischer et al. unpubl. data |
|  | *B. ormerodianum* Hermans | - | FS3149 (SZU) | Madagascar | Fischer et al. unpubl. data |
|  | *B. oxycalyx* Schltr. | EF196044.1 | - | Madagascar | [50] |
|  | *B. oxychilum* Schltr. | EF195937.1 | - | Africa | [50] |
|  | *B. pachypus* Schltr. | EF196046.1 | - | Madagascar | [50] |
|  | *B. palawanense* Garay | - | OR152_05 (SZU) | Asia | Hochschartner et al. unpubl. data |
|  | *B. paleiferum* Schltr. | EF196016.1 | - | Madagascar | [50] |
|  | *B. pantoblepharon* Schltr. | EF196048.1 | - | Madagascar | [50] |
|  | *B. patens* King ex Hook.f. | EF195938 | - | Asia | [50] |
|  | *B. perii* Schltr. | GQ862815.1 | - | South America | [135] |
|  | *B. pervillei* Rolfe ex Elliot | EF196049.1 | - | Madagascar | [50] |
|  | *B. petrae* G.A.Fischer, A.Sieder  & P.J.Cribb | EF196050.1 | - | Madagascar | [50] |
|  | *B. peyrotii* Bosser | EF196051.1 | - | Madagascar | [50] |
|  | *B. picturatum* (Lodd.) Rchb.f. | EF195939.1 | - | Asia | [50] |
|  | *B. piestoglossum* J.J.Verm. | - | OR103_98 (SZU) | Asia | Hochschartner et al. unpubl. data |
|  | *B. pileatum* Lindl. | EF195940 | - | Asia | [50] |
|  | *B. pipio* Rchb.f. | - | DSS861 (SZU) | Africa | Fischer et al. unpubl. data |
|  | *B. pipio* Rchb.f. | - | DSS887 (SZU) | Africa | Fischer et al. unpubl. data |
|  | *B. platypodum* H.Perrier | EF196052.1 | - | Madagascar | [50] |
|  | *B. pleurothallopsis* Schltr. | EF196053.1 | - | Madagascar | [50] |
|  | *B. plumosum* (Barb.Rodr.) Cogn. | EF195941.1 | - | South America | [50] |
|  | *B. polystictum* Ridl. | - | OR599_99 (SZU) | Asia | Hochschartner et al. unpubl. data |
|  | *B. porphyrostachys* Summerh. | - | DSS901 (SZU) | Africa | Fischer et al. unpubl. data |
|  | *B. prismaticum* Thouars | - | REU8 (SZU) | Madagascar, Réunion | Fischer et al. unpubl. data |
|  | *B. protectum* H.Perrier | - | OR320 (SZU) | Madagascar | Fischer et al. unpubl. data |
|  | *B. pumilum* (Sw.) Lindl. | - | AFRICA3 (SZU) | Africa | Fischer et al. unpubl. data |
|  | *B. purpureorhachis* (De Wild.) Schltr. | - | OR209 (SZU) | Africa | Fischer et al. unpubl. data |
|  | *B. pusillum var. sambiranense* G.A.Fischer & P.J.Cribb | EF195973.1 | - | Madagascar | [50] |
|  | *B. quadrialatum* H.Perrier | - | OR331 (SZU) | Madagascar | Fischer et al. unpubl. data |
|  | *B. quadrifarium* Rolfe | EF196028.1 | - | Madagascar | [50] |
|  | *B. quinquecornutum* H.Perrier | EF196060.1 | - | Madagascar | [50] |
|  | *B. radicans* F.M.Bailey | - | OR10 (SZU) | Australia | Fischer et al. unpubl. data |
|  | *B. rauhii* Toill.-Gen. & Bosser var. *andranobeense* Bosser | EF196055.1 | - | Madagascar | [50] |
|  | *B. regnellii* Rchb.f. | GQ339710.1 | - | South America | [135] |
|  | *B. renkinianum* (Laurent)  De Wild. | - | OR1394 (SZU) | Africa | Fischer et al. unpubl. data |
|  | *B. rubiginosum* Schltr. | EF196056.1 | - | Madagascar | [50] |
|  | *B. rubrum* Jum. & H.Perrier | KJ558742 | - | Madagascar | present study |
|  | *B. ruginosum* H.Perrier | - | OR1400 (SZU) | Madagascar | Fischer et al. unpubl. data |
|  | *B. rupicola* Bar.Rodr. | GQ339696.1 | - | South America | [135] |
|  | *B. saltatorium* Lindl. | - | DSS936 (SZU) | Africa | Fischer et al. unpubl. data |
|  | *B. sandersonii* (Hook.f.) Rchb.f. | - | AFRICA2 (SZU) | Africa | Fischer et al. unpubl. data |
|  | *B. sandrangatense* Bosser | EF196058.1 | - | Madagascar | [50] |
|  | *B. sandrangatense* Bosser |  | OR316 (SZU) | Madagascar | [50] |
|  | *B.* *sarcorhachis* Schltr. | EF196008.1 | - | Madagascar | [50] |
|  | *B. aff. sarcorhachis* Schltr. | EF195992.1 | - | Madagascar | [50] |
|  | *B. scaberulum* (Rolfe) Bolus | - | VD&S690 (SZU) | Africa | Fischer et al. unpubl. data |
|  | *B. schillerianum* Rchb.f. | - | Australia1 (SZU) | Australia | Fischer et al. unpubl. data |
|  | *B. schimperianum* Kraenzl. | - | DSS850 (SZU) | Africa | Fischer et al. unpubl. data |
|  | *B. schinzianum* Kraenzl. ex De Wild . & T.Durand | - | DSS1006 (SZU) | Africa | Fischer et al. unpubl. data |
|  | *B. sciaphile* Bosser | EF196059.1 | - | Madagascar | [50] |
|  | *B. senghasii* G.A.Fischer & A.Sieder | KJ558726 | - | Madagascar | present study |
|  | *B. setigerum* Lindl. | GQ339689.1 | - | South America | [135] |
|  | *B. siamense* Rchb.f. | EF195942 | - | Asia | [50] |
|  | *B. smitinandii* Seidenf. & Thorut | EF195943 | - | Asia | [50] |
|  | *B.* sp*.* | EF196003.1 | - | Madagascar | [50] |
|  | *B.* sp. ‘*11’* | EF195995.1 | - | Madagascar | [50] |
|  | *B.* sp. *‘14’* | EF196007.1 | - | Madagascar | [50] |
|  | *B.* sp. *‘15’* | EF196066.1 | - | Madagascar | [50] |
|  | *B.* sp*. ‘16’* | EF195994.1 | - | Madagascar | [50] |
|  | *B.* sp*. ‘17’* | EF196018.1 | - | Madagascar | [50] |
|  | *B.* sp. *‘21’* | EF195998.1 | - | Madagascar | [50] |
|  | *B.* sp*. ‘22’* | EF196017.1 | - | Madagascar | [50] |
|  | *B.* sp. *‘25’* | EF196000.1 | - | Madagascar | [50] |
|  | *B.* sp. *‘28A’* | EF196015.1 | - | Madagascar | [50] |
|  | *B.* sp. *‘3’* | EF196070.1 | - | Madagascar | [50] |
|  | *B.* sp. *‘6’* | EF195997.1 | - | Madagascar | [50] |
|  | *B.* sp. *‘7’* | EF196064.1 | - | Madagascar | [50] |
|  | *B.* sp. *‘9’* | EF195991.1 | - | Madagascar | [50] |
|  | *B.* sp. *‘FS957’* | EF196009.1 | - | Madagascar | [50] |
|  | *B.* sp. nov. *‘A’* | KJ558722 | - | Madagascar | present study |
|  | *B.* sp. *‘FS4365’* | - | FS4365 (SZU) | Madagascar | Fischer et al. unpubl. data |
|  | *B.* sp. nov. *‘B’* | KJ558740 | - | Madagascar | present study |
|  | *B.* sp. nov. *‘C’* | KJ558731 | - | Madagascar | present study |
|  | *B.* sp. nov. *‘*K16652’ | EF196011.1 | - | Madagascar | [50] |
|  | *B.* sp. nov*. ‘*K16657’ | EF196012.1 | - | Madagascar | [50] |
|  | *B.* sp. nov. *‘*K16935’ | EF196013.1 | - | Madagascar | [50] |
|  | *B.* sp. nov. *aff. saltatorium* | - | BS120 (SZU) | Africa | Fischer et al. unpubl. data |
|  | *B. aff. sphaerobulbum* H.Perrier | EF195950.1 | - | Madagascar | [50] |
|  | *B. steyermarkii* Foldats | GQ339688.1 | - | South America | [135] |
|  | *B. subclavatum* Schltr. | EF196006.1 | - | Madagascar | [50] |
|  | *B. subligaculiferum* J.J.Verm. | - | DSS1486 (SZU) | Africa | Fischer et al. unpubl. data |
|  | *B. sulfureum* Schltr. | EF196071.1 | - | Madagascar | [50] |
|  | *B. sumatranum* Garay, Hamer & Siegerist | - | OR150 (SZU) | Asia | Hochschartner et al. unpubl. data |
|  | *B. teimosense* E.C.Smidt & Borba | GQ339703.1 | - | South America | [135] |
|  | *B. teretifolium* Schltr. | - | DSS1055 (SZU) | Africa | Fischer et al. unpubl. data |
|  | *B. tetragonum* Lindl. | - | DSS872 (SZU) | Africa | Fischer et al. unpubl. data |
|  | *B. therezienii* Bosser | - | FS4071 (SZU) | Madagascar | Fischer et al. unpubl. data |
|  | *B. trifarium* Rolfe | EF196072.1 | - | Madagascar | [50] |
|  | *B. turkii* Bosser & P.J.Cribb | EF196073.1 | - | Madagascar | [50] |
|  | *B. vanum* J.J.Verm. | - | DSS875 (SZU) | Africa | Fischer et al. unpubl. data |
|  | *B. variegatum* Thouars | EF196074.1 | - | Madagascar | [50] |
|  | *B. variifolium* Schltr. | - | FS2532 (SZU) | Madagascar | Fischer et al. unpubl. data |
|  | *B. ventriosum* H.Perrier | EF196075.1 | - | Madagascar | [50] |
|  | *B. vestitum* Bosser | - | OR310 (SZU) | Madagascar | Fischer et al. unpubl. data |
|  | *B. vestitum* Bosser var.  *meridionale* Bosser | EF196068.1 | - | Madagascar | [50] |
|  | *B. weddellii* (Lindl.) Rchb.f. | GQ339713.1 | - | South America | [135] |
|  |  |  |  |  |  |
| *Dendrobium* | |  |  |  |  |
|  | *D. brymerianum* Rchb.f. | AF362036.1 | - | Asia | [134] |
|  | *D. crepidatum* Lindl. & Paxton | AF355574.2 | - | Asia | [134] |
|  | *D. crumenatum* Swartz | AF521608.1 | - | Asia | [134] |
|  | *D. devonianum* Paxton | EF629322.1 | - | Asia | [134] |
|  | *D. ellipsophyllum* T.Tang & F.T.Wang | AF362033.1 | - | Asia | [134] |
|  | *D. equitans* Kraenzl. | AF521609.1 | - | Asia | [133] |
|  | *D. furcatopedicellatum* Hayata | AF521611.1 | - | Asia | [133] |
|  | *D. gratiosissimum* Rchb.f. | AF311780.1 | - | Asia | [134] |
|  | *D. linawianum* Rchb.f. | AF521613.1 | - | Asia | [133] |
|  | *D. nobile* Lindl. | AF362045.1 | - | Asia | [134] |
|  | *D. officinale* Kimura & Migo | AF311776.1 | - | Asia | [134] |

^*^Acronym of herbarium (SZU) in which voucher specimens are deposited is in parentheses.
