# Supplementary material for: Multiple independent origins of auto-pollination in tropical orchids (Bulbophyllum) in light of the hypothesis of selfing as an evolutionary dead end
Source: BMC Evol Biol. 2015 Sep 16;15:192. doi: 10.1186/s12862-015-0471-5 (PMC4574068; doi:10.1186/s12862-015-0471-5)
Supplement: Additional file 12: — Age estimates of the family Orchidaceae and various subclades as compared to previous ones. (DOCX 17 kb) [file 12862_2015_471_MOESM12_ESM.docx]

**Additional file 12**

**Gamisch et al. “Multiple independent de novo origins of auto-pollination in tropical orchids (*Bulbophyllum*) in light of the hypothesis of selfing as an evolutionary dead end”**

**Additional file 12: Age estimates of the family Orchidaceae and various subclades as compared to previous ones.** Median and 95% highest posterior density (HPD) crown and stem group ages (in million years ago) estimated for the family Orchidaceae and various subclades as compared to previous estimates by [133], excepting the newly estimated stem age of *Dendrobium*/*Bulbophyllum*.

| Age/clade | | Median age (95% HPD) | |
| --- | --- | --- | --- |
|  |  | Gustaffson et al.[133] | This study |
| Crown group ages | |  |  |
|  | Family Orchidaceae | 77 (63–92) | 84.3 (68–101.7) |
|  | Subfamily Apostasioideae | 41 (23–61) | 39.9 (23.6–58) |
|  | Subfamily Vanilloideae | 57 (43–72) | 63.1 (48.4–78.2) |
|  | Subfamily Cypripedioideae | 33 (19–50) | 32.7 (20.9–46.7) |
|  | Subfamily Orchidoideae | 53 (42–64) | 55.3 (44.1–67.4) |
|  | Subfamily Epidendroideae | 49 (38–62) | 46.7 (36.5–58.1) |
|  | 'Higher Epidendroids' | 39 (31–49) | 37.4 (30–45.8) |
|  | Subtribe Goodyerinae | 32 (23–41) | 34.4 (25.8 –43.7) |
|  | *Dendrobium* | 23 | 22 (20–27.4) |
|  | *Earina* | 16 | - |
| Stem group ages | |  |  |
|  | Family Orchidaceae | 109 | 109.4 (95.5–120) |
|  | Subfamily Apostasioideae | 77 | 84.3 (68–101.7) |
|  | Subfamily Cypripedioideae | 71 | 77 (62–93) |
|  | Subfamily Vanilloideae | 69 | 74.5 (59.6–89.9) |
|  | Subfamily Orchidoideae | 61 | 65.5 (52.2–79.4) |
|  | Subfamily Epidendroideae | 61 | 65.5 (52.2–79.4) |
|  | 'Higher Epidendroids' | 45 | 42.6 (33.7–52.7) |
|  | Subtribe Goodyerinae | 38 | 40.1 (31.2–49.7) |
|  | *Dendrobium/Agrostophyllum-Earina* | 32 | - |
|  | *Dendrobium/Bulbophyllum* | - | 29.3 (23.3–37) |
