# Supplementary material for: Multiple independent origins of auto-pollination in tropical orchids (Bulbophyllum) in light of the hypothesis of selfing as an evolutionary dead end
Source: BMC Evol Biol. 2015 Sep 16;15:192. doi: 10.1186/s12862-015-0471-5 (PMC4574068; doi:10.1186/s12862-015-0471-5)
Supplement: Additional file 13: — Infrageneric age estimates of the genus Bulbophyllum. (DOCX 17 kb) [file 12862_2015_471_MOESM13_ESM.docx]

**Additional file 13**

**Gamisch et al. “Multiple independent de novo origins of auto-pollination in tropical orchids (*Bulbophyllum*) in light of the hypothesis of selfing as an evolutionary dead end”**

**Additional file 13: Infrageneric age estimates of the genus *Bulbophyllum*.** Median and 95% highest posterior density (HPD) age estimates (in million years ago) for key nodes of the genus *Bulbophyllum* identified in [Additional file 8]. Age estimates for Madagascan *Bulbophyllum* clade C are in bold.

| Node | Clade | Median age | 95% HPD |
| --- | --- | --- | --- |
| A | *Bulbophyllum* (crown) | 15.65 | 9.83–22.48 |
| B1 | Asian-Australian subclade 1 | 12.55 | 5.51–18.21 |
| B2 | Asian-Australian subclade 2 | 10.91 | 6.5–16.34 |
| B3 | Asian-Australian subclade 3 | 8.84 | 4.87–13.45 |
| B4 | Asian subclade 4 | 4.82 | 2.27–8.54 |
| B5 | Asian subclade 5 | 3.14 | 1.51–5.42 |
| C | Madagascan-African-Neotropic clade (crown) | 12.26 | 7.51–17.77 |
| D | African-Neotropic clade (crown) | 9.68 | 5.85–14–2 |
| E | African clade (crown) | 7.24 | 3.81–11.32 |
| E1 | African subclade 1 | 4.58 | 2.51–7.17 |
| E2 | African subclade 2 | 2.28 | 0.82–4.36 |
| E3 | African subclade 3 | 1.97 | 0.88–3.45 |
| F | Neotropic clade (crown) | 7.24 | 4.28–10.79 |
| F1 | Neotropic subclade 1 | 5.73 | 3.29–8.65 |
| F2 | Neotropic subclade 2 | 4.85 | 2.81–7.44 |
| F3 | Neotropic subclade 3 | 2.77 | 1.38–4.57 |
| G | Madagascan clade (crown) | 10.55 | 6.37–15.46 |
| G1 | Madagascan subclade 1 | 5.65 | 3.16–8.56 |
| **G2** | **Madagascan subclade 2** | **5.32** | **2.66–8.5** |
| G3 | Madagascan subclade 3 | 4.9 | 2.16–7.03 |
| G4 | Madagascan subclade 4 | 2.26 | 0.88–4.36 |
| G5 | Madagascan subclade 5 | 1.62 | 0.84–2.7 |
